# Indole-Induced Activities of β-Lactamase and Efflux Pump Confer Ampicillin Resistance in *Pseudomonas putida* KT2440

**DOI:** 10.3389/fmicb.2017.00433

**Published:** 2017-03-14

**Authors:** Jisun Kim, Bora Shin, Chulwoo Park, Woojun Park

**Affiliations:** Laboratory of Molecular Environmental Microbiology, Department of Environmental Science and Ecological Engineering, Korea University, SeoulKorea

**Keywords:** indole, ampicillin, antibiotics, bacteria, resistance, efflux pump, *Pseudomonas*

## Abstract

Indole, which is widespread in microbial communities, has received attention because of its effects on bacterial physiology. *Pseudomonas putida* and *Pseudomonas aeruginosa* can acquire ampicillin (Amp) resistance during growth on indole-Amp agar. Transcriptome, mutant, and inhibitor studies have suggested that Amp resistance induced by indole can be attributed to increased gene expression of *ttgAB* encoding two genes of RND-type multidrug efflux operons and an *ampC* encoding β-lactamase. Expression, enzyme activities, and mutational analyses indicated that AmpC β-lactamase is important for acquiring Amp resistance of *P. putida* in the presence of indole. Here, we show, for the first time, that volatile indole increased Amp-resistant cells. Consistent with results of the volatile indole assay, a low concentration of indole in liquid culture promoted growth initially, but led to mutagenesis after indole was depleted, which could not be observed at high indole concentrations. Interestingly, *ttgAB* and *ampC* gene expression levels correlate with the concentration of indole, which might explain the low number of Amp-mutated cells in high indole concentrations. The expression levels of genes involved in mutagenesis, namely *rpoS*, *recA*, and *mutS*, were also modulated by indole. Our data indicates that indole reduces Amp-induced heterogeneity by promoting expression of TtgABC or MexAB-OprM efflux pumps and the indole-induced β-lactamase in *P. putida* and *P. aeruginosa*.

## Introduction

Indole has received a great deal of attention because of its broad range of effects on bacterial physiology, including biofilm formation (Lee et al., 2007; Kim and Park, 2013; Kim et al., 2015), quorum sensing (Chu et al., 2012; Sabag-Daigle et al., 2012; Kim and Park, 2013), virulence (Lee et al., 2009; Chu et al., 2012; Nikaido et al., 2012), spore formation (Stamm et al., 2005), plasmid stabilization (Field and Summers, 2012), and antimicrobial resistance (hereafter, AMR) (Hirakawa et al., 2005; Lee H.H. et al., 2010; Nikaido et al., 2012; Vega et al., 2012, 2013). Enteric bacteria produce indole from tryptophan by the action of tryptophanase (TnaA) (Yanofsky et al., 1991). TnaA can convert tryptophan into indole, pyruvate, and ammonia (Newton and Snell, 1965). Indole is transported or diffused outside of cells, where indole concentrations commonly reach 1–2 mM, and sometimes up to 5 mM, in the stationary growth phase (Li and Young, 2013). Furthermore, a recent report suggested that the intracellular concentration of indole could increase transiently in rapidly growing cells and reach 60 mM during the stationary phase (Gaimster et al., 2014). Thus, indole non-producing bacteria can be exposed to various amounts of indole excreted by indole-producing bacteria. However, research on how exogenous indole is transported into different bacterial cells, the effects of indole on various cellular processes, and molecular mechanisms for sensing indole remain to be established. The physiological roles of indole may differ depending on environmental conditions (e.g., nutrient availability and temperature), indole concentration, and strain traits (e.g., indole-producing ability). For example, biofilm formation is enhanced by indole in *Pseudomonas* and *Agrobacterium*, but not in *Escherichia coli* (Lee et al., 2009; Kim et al., 2013). Large amounts of indole inhibit growth and cell division because indole can act as a proton ionophore (Chimerel et al., 2012). When indole is imported across a cellular membrane, the electrochemical potential and adenosine triphosphate (ATP) concentration inside of the cell can decrease and the NADH/NAD^+^ ratio is modulated (Piñero-Fernandez et al., 2011; Chimerel et al., 2012; Kim et al., 2013).

Antibiotic-susceptible bacteria can acquire AMR by upregulating expression of genes involved in stress defenses such as multidrug efflux pumps (Fernández and Hancock, 2012; Nikaido et al., 2012). It has been reported that indole increases AMR by activating defense systems, promoting the formation of persister cells (Hirakawa et al., 2005; Lee H.H. et al., 2010; Vega et al., 2012; Molina-Santiago et al., 2014). Indole can increase the expression of genes encoding multidrug exporters: *mdtAE*, *cusB*, *emrK*, and *yceL* in *E. coli* (Hirakawa et al., 2005), *acrAB* in *Salmonella enterica* (Nikaido et al., 2012), *ttgGHI* in *P. putida* (Molina-Santiago et al., 2014), *emrA, norm* and *Atu25521* in *A. tumefaciens* (Lee et al., 2015a). Shikimate kinase (encoded by *aroK*) can produce aromatic metabolites, including indole (Sulavik et al., 1995), and can upregulate the CpxAR two-component regulatory system, resulting in transcriptional activation of genes related to multiple AMR such as the *marRAB* operon, which facilitates production of multidrug efflux pumps in *E. coli* (MdtABC, AcrAB, and EmrAB) (Hirakawa et al., 2005; Weatherspoon-Griffin et al., 2014). These findings suggest that aromatic metabolites, including indole, may perform crucial functions in the response to antibiotics by adjusting regulatory cascades.

Indole-mediated antibiotic tolerance may result from the induction of genes that participate in oxidative stress defenses (*oxyS* and *dps*, which belong to the OxyR regulon) and the phage shock response in *E. coli* and *S.* Typhimurium (Vega et al., 2012, 2013). However, no significant alterations in the expression of oxidative-stress defense genes in response to indole have been reported in *P. putida*, which also shows an ampicillin (Amp) resistance phenotype in the presence of exogenous indole, and superoxide production has not been detected in the presence of indole (Kim et al., 2013). Thus, AMR enhanced by indole cannot be completely understood based on oxidative stress defense mechanisms alone.

Here, we show that exogenous indole can increase the AMR of indole non-producing *P. putida* and *P. aeruginosa*. This resistance was acquired through long-term exposure to indole in either liquid or solid media. Interestingly, aerial exposure to volatile indole can also enhance AMR by increasing the number of mutated cells when cells are far from the indole source, whereas Amp-resistant cells near the source show a low number of mutated cells. Our data suggest that acquisition of resistance in the presence of indole can affect Amp-induced heterogeneity of cells through the action of TtgABC or MexAB-OprM efflux pumps and β-lactamase in *P. putida* and *P. aeruginosa*.

## Materials and Methods

### Bacterial Strains and Culture Conditions

Bacterial strains and primers used in this study are shown in Supplementary Table S1. *P. putida* KT2440 and *P. aeruginosa* strains were grown at 30°C and 37°C in Luria-Bertani (LB) and modified M9 media [Na_2_HPO_4_7H_2_O (6.8 g/l), KH_2_PO_4_ (3 g/l), NaCl (0.5 g/l), NH_4_Cl (1 g/l), MgSO_4_ (2 mM), and CaCl_2_ (0.1 mM)] (Sambrook et al., 1989) containing 10 mM glucose and 10 mM succinate (Lee Y. et al., 2010) with aeration and shaking. Reagents in media were purchased from Sigma (USA). Growth was monitored by measuring the optical density of cultures at 600 nm (OD_600_) using a biophotometer (Eppendorf, Germany) or by counting CFU.

### Chemical Treatments

The following chemicals were purchased from Sigma (USA): indole, phenyl-arginine-beta-naphthylamide (PAβN), indole-acetic acid, tryptophan, tetracycline (Tet), carbenicillin (Car), rifampicin (Rif), chloramphenicol (Chl), norfloxacin (Nor), and kanamycin (Kan). Ticarcillin (Tic) and apramycin (Apr) were purchased from RPI. Ampicillin (Amp) was purchased from AMRESCO. Gentamicin (Gen) was purchase from Gibco. Indole-acetic acid, Amp, Car, Nor, Kan, Tic, Apr and Gen were dissolved in distilled water. Indole, tryptophan, Tet, and Chl were dissolved in ethanol. PAβN was dissolved in 0.5% dimethyl sulfoxide (DMSO), and Rif was dissolved in methanol. Solvent effects can generally be ignored, because they were not significantly affected by changes in the solvent.

### Determination of the Minimal Inhibitory Concentrations

The MICs were determined using two fold dilution method (Irith et al., 2008). MICs were defined as the antibiotic concentration that inhibited growth after 24 h of incubation in LB liquid medium at 30°C. Overnight cultures were collected and washed two times with PBS. Approximately 10^6^ CFU/ml cells were inoculated into 96-well microtiter plates containing fresh LB medium and antibiotics. Microtiter plates were incubated for 24 h at 30°C.

### Susceptibility Tests

*Pseudomonas putida* KT2440 and *P. aeruginosa* strains were grown in LB liquid media with shaking at 30°C and 37°C, respectively. The stationary growth phase cells were diluted 100 fold in fresh media and incubated until the cells reach an OD_600_ of the exponential phase (OD_600_ of ~0.4). The exponentially growing cells were harvested by centrifugation and washed twice with phosphate-buffered saline (PBS). Cells were inoculated into PBS at approximately 10^7^ CFU/ml and serially diluted. Each dilution was spotted on an LB agar plate and incubated at the optimal temperature for the specific strain for 24 h. Agar plates were supplemented with indole, antibiotics, or both indole and antibiotics. To test volatile indole-mediated alterations in Amp resistance, indole was provided from an emitting source (25 μ moles on a paper disk) of a small plate placed inside of a square plate separated from the medium.

### The *ampC* Mutant Construction

The primers used in this study are listed in Supplementary Table S1. A 311 bp fragment of the internal region of the *ampC* gene was amplified using the KT *ampC* SC-F and KT *ampC* SC-R primers. The polymerase chain reaction (PCR) product for the *ampC* mutant was digested with the *Eco*RI and *Kpn*I restriction enzymes. Fragment was subsequently inserted into a pVIK112 vector via ligation. The constructed plasmids were then transformed into the *E. coli* S17-1 λ pair. Conjugation was performed using the biparental filter mating method.

### β-Lactamase Activity Assay

β-lactamase activity was measured by hydrolysis of nitrocefin (O’Callaghan et al., 1972; Cavallari et al., 2013; Liu et al., 2016). Cells were grown to the exponential phase at 30°C with aeration. The cells were then treated with or without Amp or indole for 3 h. Following incubation, 5 ml culture was pelleted, washed with PBS (pH 7.0), and resuspended in the same buffer. Samples were placed on ice and then lysed by sonication, centrifuged, and supernatants were collected. The reaction was initialized by adding cell lysate contained β-lactamase to the reaction mixture containing 25 μg nitrocefin (abcam) and PBS (pH 7.0), and the total volume for each reaction was 1 ml. Enzymatic reaction was performed at 30°C for 10 min, and the change at 486 nm was measured over 1 min on a UV/visible spectrophotometer. The extinction coefficient for degraded nitrocefin was 20.5 mM^-1^ cm^-1^. Enzyme activity was measured in nmol min^-1^ mg^-1^ nitrocefin hydrolyzed.

### Microarray Analysis

*Pseudomonas putida* KT2440 cells were grown overnight in LB medium and then diluted 100 fold. When the diluted cells reached the exponential phase (OD_600_ ~0.4), the cells were collected and washed two times with PBS. Appropriate dilutions of the cells were spread on LB plates containing 1 mM indole, 50 μg/ml Amp, or both 1 mM indole and 50 μg/ml Amp. After 12 h of incubation at 30°C, cells were collected from the plates, which contained approximately 100 colonies per plate. Total RNA was isolated using the RNAprotect Bacteria Reagent (Qiagen, Valencia, CA, USA) and RNeasy Mini Kit (Qiagen, Valencia, CA, USA) according to the manufacturer’s instructions. The following procedure was conducted, as previously described Kim et al. (2013). Genes that showed increases of more than two fold (upregulated genes) or decreases more than 0.5 fold (downregulated genes) were selected. The microarray data were deposited in the National Center for Biotechnology Information (NCBI) GEO site (accession number GSE 86617). cDNA probes for the cDNA microarray analysis were prepared by reverse transcription of total RNA (50 μg) in the presence of aminoallyl-dUTP and 6 μg of random primers (Invitrogen, Carlsbad, CA, USA) for 3 h. The cDNA probes were cleaned using a Microcon YM-30 column (Millipore, Billerica, MA, USA), followed by coupling to the Cy3 dye (for reference) or Cy5 dye (for test samples) (Amersham Biosciences Pharmacia, Amersham, UK). The dried Cy3- or Cy5-labeled cDNA probes were then resuspended in hybridization buffer containing 30% formamide (v/v), 5 × saline-sodium citrate, 0.1% sodium dodecyl sulfate (SDS) (w/v), and 0.1 mg/mL salmon sperm DNA. The Cy3- or Cy5-labeled cDNA probes were mixed and hybridized onto a microarray slide. The hybridization images on the slides were scanned using an Axon 4000B microarray scanner (Axon Instruments, Union City, CA, USA) and analyzed with GenePix Pro software (version 3.0, Axon Instruments) to determine the gene expression ratios (control *versus* test samples).

### Gene Expression Analysis by Northern Blotting

*Pseudomonas putida* KT2440 cells were grown overnight in LB medium and then diluted 100 fold. Appropriate dilutions of exponentially growing cells were spread on LB plates containing indole, Amp, or both indole and Amp. After 36 h of incubation at 30°C, cells were collected from the plates, and total RNA was isolated using an RNeasy Mini Kit according to the manufacturer’s instructions. Northern blot analysis was then performed as described previously (Kim and Park, 2013). Samples of total RNA (2.5 μg) were loaded onto denaturing agarose gels containing 0.25 M formaldehyde, separated, and then stained with ethidium bromide to visualize 23S and 16S rRNA. The fractionated RNA was transferred to nylon membranes (Schleicher and Schuell, Germany) using a TurboBlotter (Schleicher and Schuell, Germany). The amount of mRNA was determined by hybridizing the membrane with a specific ^32^P-labeled probe (Takara, Japan), prepared by PCR amplification with the respective primer pairs. Autoradiography was conducted using an IP plate (Fujifilm, Japan) and Multiplex Bio-Imaging System (Fujifilm, Japan).

### Determination of the Percentage of Mutated Cells

Overnight cultures were collected and washed two times with PBS. Approximately 10^5^ CFU/ml cells were inoculated into fresh LB medium containing 100 μg/ml Amp and indole (0, 50, 100, 250, 500, and 1000 μM) and incubated for 30 h at 30°C and 200 rpm/min. To verify whether adaptive transitional resistance occurred or not, cells were transferred to fresh media and incubated before determining of the percentage of mutated cells. Cells were collected and washed twice with PBS to remove remaining Amp and indole and approximately 10^6^ CFU/ml cells were inoculated into fresh LB medium and incubated for 24 h at 30°C. Appropriate dilutions of the cells were spread on LB plates containing 50 μg/ml Rif or 200 μg/ml Amp. The total number of CFUs was determined on LB agar plates, with colonies counted after 24 h of incubation at 30°C. To measure the percentage of mutated cells grown on agar plates, 10^5^ CFU were spotted on LB agar plates containing 75 μg/ml Amp. Indole was provided from an emitting source (25 μ moles in paper disk) of a small plate placed inside of a square plate separated from the medium. After 24 h of incubation at 30°C, cells were sampled from each region, based on the distance from the indole source, and incubated in fresh LB medium for 24 h at 30°C. And then, incubated cells were diluted and spread on Amp- or Rif-containing LB plates. The total number of CFUs was determined on LB agar plates, with colonies counted after 24 h of incubation at 30°C. The percentage of mutated cells was determined based on the relative percentage of CFU/ml [(CFUs obtained from the antibiotic plate/total number of CFUs obtained from the LB plate)*100].

### Gene Expression Analysis by Quantitative Reverse Transcriptase-PCR (qRT-PCR)

Approximately 10^5^ CFU/ml *P. putida* KT2440 cells grown overnight were inoculated into fresh LB medium containing 100 μg/ml Amp and indole (0, 50, 100, 250, 500, and 1000 μM) and incubated for 8 h at 30°C. Total RNA was isolated using an RNeasy Mini Kit according to the manufacturer’s instructions, and cDNA was synthesized from 1 μg of RNA used as a template with primers for the target gene. The PCR mixture contained 12.5 μl of iQ SYBR Green Supermix (Bio-Rad, Hercules, CA, USA), 1 μl of each primer (0.5 μM) (Supplementary Table S1), and 2 μl of cDNA in a total volume of 25 μl. The PCR conditions were 95°C for 3 min, followed by 40 cycles of 45 s at 95°C, 45 s at 60°C, and 45 s at 72°C. To normalize the expression of each gene, the expression level of 16S rDNA was quantified with primers used previously (Watanabe et al., 2001). The results were determined from experiments performed in triplicate.

## Results

### Indole Can Increase AMR in *P. putida* KT2440 and *P. aeruginosa* PAO1

**FIGURE 1:**
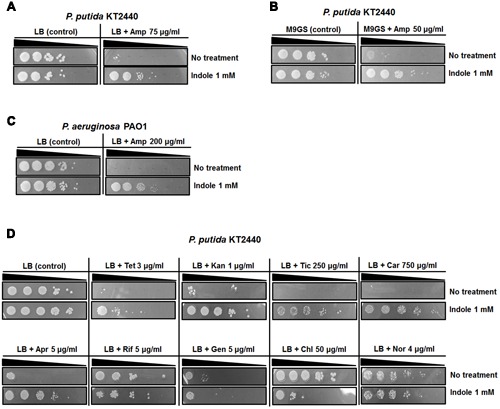
**Acquisition of AMR by *Pseudomonas* species in response to indole.** Amp susceptibility test with or without 1 mM indole in *P. putida* KT2440 **(A)** and *P. aeruginosa* PAO1 **(C)**. The effects of indole on the acquisition of Amp resistance in M9GS medium (M9 medium supplemented with 10 mM glucose and 10 mM succinate) **(B)**. **(D)** Various antibiotics susceptibility test with or without indole in *P. putida* KT2440. The exponentially growing cells were harvested and washed twice with phosphate-buffered saline (PBS). Approximately 10^7^ CFU/ml cells were inoculated into PBS and serially diluted. Each dilution of the solution was spotted on plates and incubated at the optimal temperature for the species for 24 h. Tet, tetracycline; Kan, kanamycin; Tic, ticarcilllin; Car, carbenicillin; Apr, apramycin; Rif, rifampicin; Gen, gentamicin; Chl, chloramphenicol; Nor, norfloxacin.

Indole has been known to increase AMR by enhancing persister cell formation in *E. coli* (Lee H.H. et al., 2010; Vega et al., 2012, 2013). To examine whether *P. putida* KT2440 showed increased survived cells with indole treatments, survived cells were quantified after cells were exposed to a high concentration of Amp (200 μg/ml) in the presence of indole. We did not observe that indole induces resistance in *P. putida* KT2440 (Supplementary Figure S1). Neither the simultaneous addition of indole and Amp, nor indole pretreatment before exposure to Amp led to increased numbers of survived cells. We determined the minimum inhibitory concentrations (MICs) of various antibiotics in *P. putida* KT2440 and found that those MICs were identical in the absence or presence of 1 mM indole except for ticarcillin MIC (Supplementary Table S2). However, indole supplied at the beginning of growth increased Amp-resistant cells in LB agar (**Figure 1A**). We also verified the acquisition of Amp resistance in different minimal media (**Figure 1B**). Resistance to Amp in the presence of indole also appeared in *P. aeruginosa* PAO1, *Acinetobacter oleivorans* DR1, and *E. coli* O157:H7, regardless of their ability to produce indole (**Figure 1C**, Supplementary Figure S2A). Indole also increased resistance to tetracycline, kanamycin, other β-lactam antibiotics (ticarcilllin and carbenicillin), and apramycin in LB agar plates (**Figure 1D**, Supplementary Figure S2B). But, the acquisition of resistance was not observed with rifampicin, gentamicin, chloramphenicol, and norfloxacin resistance in the presence of indole (**Figure 1D**). Therefore, it seems likely that long-term exposure to indole can enhance AMR in many bacterial species. For a more detailed study of the effects of indole on AMR, *Pseudomonas* species and the β-lactam antibiotic, Amp, were chosen as a model species and drug, respectively.

### Indole Triggers a Broad Range of Transcriptional Responses during Long-term Exposure to Amp

To identify genes that are important for Amp resistance in *P. putida* KT2440 on indole-agar plates, a microarray analysis was conducted. Detailed procedures for the preparation of cells are shown in Supplementary Figure S3. Two genes, *trpA* and *trpB* (encoding tryptophan synthase), were highly upregulated by continuously supplied indole, indicating that the tryptophan pathway is induced by indole (Supplementary Figure S4, Supplementary Table S3). Several oxygenases and cytochrome C oxidases required for degradation of indole in non-indole-producing bacteria (Lee and Lee, 2010; Lee et al., 2015b) were highly expressed in the presence of indole (Supplementary Table S4). Ring-cleaving dioxygenase (encoded PP_3328) was increased over 10 fold by indole, indicating that this gene might be important to modifying or degrading indole in *P. putida*. Genes involved in tryptophan metabolism and oxidation were upregulated by indole, which suggested that indole was degraded under our tested conditions. Genes responsible for the tricarboxylic acid cycle (TCA cycle) and chaperones/proteases that immediately responded to indole during a 10-min treatment in liquid media, as shown in our previous report (Kim et al., 2013), showed mild upregulation with long-term exposure to indole in the transcriptome data, suggesting that the effects of indole were not very different between liquid and plate growth conditions (Supplementary Table S3). Among the genes involved in β-lactam resistance (Nakae et al., 1999; Quale et al., 2006; Kong et al., 2010), genes encoding resistance nodulation cell division (RND) efflux pumps/transporters (*ttgABC, acrB2, and acrB3*) and bacterial secretion systems, specifically, were increased by indole plus Amp or only indole (Supplementary Tables S5–S7), suggesting that they might contribute primarily to the acquisition of indole-induced Amp resistance. Two genes (PP_1239 and PP_5084) encoding β-lactamase and penicillin-binding protein were upregulated 1.5 fold by indole with Amp; however, most of genes encoding β-lactamases and penicillin binding proteins were not expressed even in Amp alone, probably because the concentration of Amp is a factor controlling the induction of these genes (Supplementary Table S5). Expression of almost 40 genes were increased more than 1.5 fold following treatment for a short time with 1 mM indole in our previous microarray study (Kim et al., 2013). However, 189, 193, and 162 genes were upregulated by indole, Amp, and indole plus Amp, respectively, indicating that both chemicals can cause a broad range of transcriptional responses during long-term exposure of *P. putida*.

### RND-Type Efflux Pumps and β-Lactamases Are Essential for the Acquisition of Indole-Mediated AMR

**FIGURE 2:**
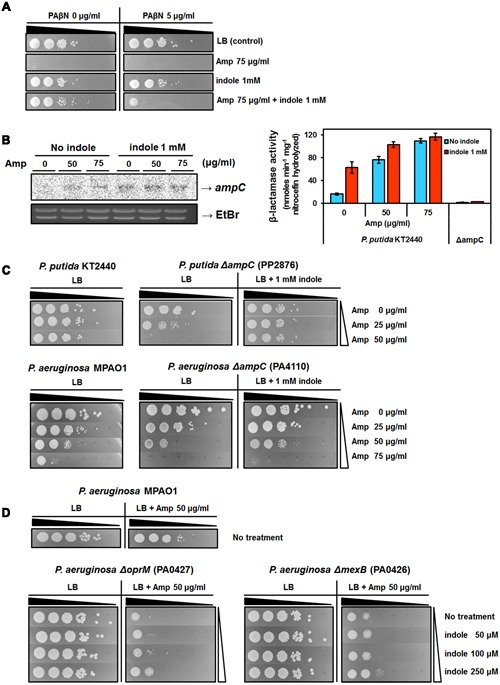
**Contribution of efflux pumps and β-lactamase to AMR induced by indole. (A)** The effects of PAβN on Amp resistance induced by indole. PAβN (5 μg/ml was added to the agar plate. **(B)** Expression of the *ampC* gene and β-lactamase activity in the presence of indole and Amp during growth. Total RNA was isolated from cultures after 36 h of incubation with 50 or 75 μg/ml Amp or 1 mM indole or both of these. Exponentially growing cells were treated with Amp or indole for 3 h. Following incubation, β-lactamase activity was measured. Enzyme activity was measured in nmol min^-1^ mg^-1^ nitrocefin hydrolyzed. **(C)** Amp susceptibility test in *P. putida ΔampC* and *P. aeruginosa ΔampC* (PA4110). Exponentially growing cells were serially diluted, and each dilution of the solution was spotted on plates and incubated for 24 h. **(D)** Amp susceptibility test in *P. aeruginosa ΔoprM* (PA0427) and *ΔmexB* (PA0426) and *P. aeruginosa* MPAO1.

According to our transcriptome analysis and previous reports (Kim et al., 2013), genes encoding efflux pumps and transporters were upregulated by indole (Supplementary Tables S5, S6). To check the importance of RND-type efflux pumps in AMR acquired in response to indole treatment, phenyl-arginine-beta-naphthylamide (PAβN), which is a well-studied broad-spectrum RND-type efflux pump inhibitor (Lamers et al., 2013; Li et al., 2015; Opperman and Nguyen, 2015), was used. The MIC of PAβN was 256 μg/ml in liquid LB media and over 100 μg/ml in LB agar plate, but *P. putida* KT2440 experienced severe growth defects with over 8 μg/ml of PAβN. A low concentration of PAβN, without any toxic effects, reduced the acquisition of indole-mediated AMR (**Figure 2A**). Our data demonstrated that indole increased AMR through the action of RND-type efflux pumps in *P. putida* KT2440. A wide range of β-lactamases in *Pseudomonas* confer resistance to β-lactam antibiotics (Thomson and Bonomo, 2005; Zeng and Lin, 2013). In our transcriptome analysis, the expression of several genes encoding β-lactamases and penicillin binding proteins were increased by indole (Supplementary Table S5). However, *ampC*, encoding one of the best characterized β-lactamases that confers ampicillin resistances to many bacteria (Zeng and Lin, 2013), was not induced by any conditions assessed in this study. We checked the expression level of *ampC* (PP_ 2876) by Northern blot assay and β-lactamase activity with different concentrations of Amp and showed that the expression of *ampC* increased with long exposure to Amp, and, surprisingly, only the addition of indole enhanced *ampC* expression and β-lactamase activity in *P. putida* (**Figure 2B**). The *ampC* mutant showed low level of β-lactamase activity in *P. putida* and increased Amp sensitivity compared to its parental strain in *P. putida* and *P. aeruginosa* (**Figure 2C**). Thus, the expression of several β-lactamases, including *ampC*, might be essential for AMR induction by indole, along with efflux pumps.

### Indole May Modulate the Better Survival in Amp-Susceptible *P. aeruginosa* Mutants

To confirm the contribution of genetic factors identified by our transcriptome analysis to indole-induced AMR, Amp susceptibility tests using *P. aeruginosa* mutants were performed (Supplementary Figure S5, Supplementary Table S8). Contrary to our expectation, most mutant strains showed increased Amp resistance with exposure to indole. Three genes (PA1413 encoding the LysR family transcriptional regulator, PA4353, and PA4284 exodeoxyribonuclease V beta subunit) are highly expressed by indole plus Amp and conserved among *Pseudomonas* species. These deletion mutants showed high sensitivity to Amp (Supplementary Figure S6), which indicated that these genes could contribute to Amp resistance in *P. aeruginosa*. The VgrG protein (encoded by PA1511), which participates in type VI secretion systems (T6SS) in *P. aeruginosa*, was highly expressed in indole plus Amp conditions (Supplementary Table S7). However, the mutant strain survived in the presence of high concentrations of Amp (Supplementary Figure S7). *Pseudomonas* strains can carry several copies of *vgrG* and T6SS clusters in their genomes (Barret et al., 2011), and only three VgrG proteins (encoded by PA0091, PA0095, and PA2685) have been characterized (Hachani et al., 2011). Disruption of the PA1511 gene can result in Amp resistance, providing evidence to clarify the function of novel and uncovered T6SS clusters. Interestingly, three mutant strains, RND transporter mutants (*ΔoprM* and *ΔmexB*) and a β-lactamase mutant (*ΔampC*), lost indole-induced AMR (**Figures 2C,D**). However, these mutants also acquired indole-induced Amp resistance with an adjustment in the concentration of indole (Supplementary Figures S6, S7). Thus, effective concentrations of indole might contribute to increased AMR in bacteria, even in strains with different susceptibilities. Therefore the acquisition of resistance in response to indole could not fully be explained by the action of efflux pumps or β-lactamase alone in *P. aeruginosa* PAO1.

### Aerial Exposure to Indole Promotes AMR and Cellular Heterogeneity

**FIGURE 3:**
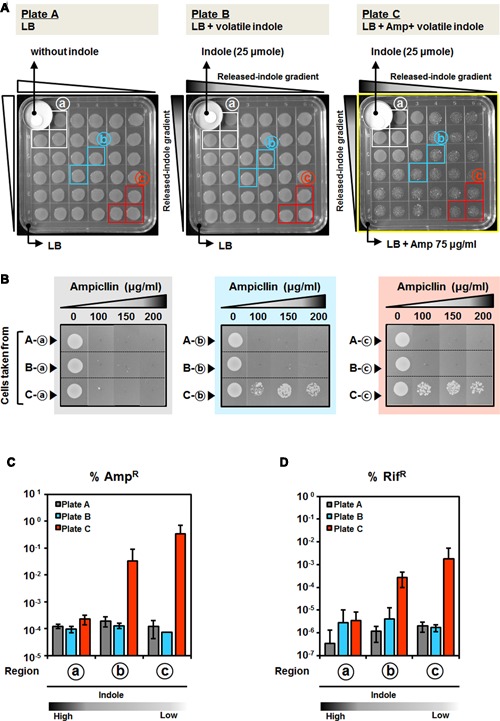
**Antimicrobial resistance induced by airborne indole without physical interaction. (A)** AMR induced by indole at a distance. Exponentially growing cells were spotted on LB plates containing 75 μg/ml Amp. Indole was provided without physical interaction with the emitting source (25 μ moles on a paper disk) of a small plate placed inside of a square plate. After 24 h of incubation at 30°C, cells were taken from each region of the plate (indicated by circles a, b, and c in **Figure 3A**), and divided based on their distances from the indole sources, and their susceptibility to Amp **(B)**, percentage of Amp-resistant (Amp^R^) **(C)**, and percentage of Rif-resistant (Rif^R^) **(D)** were determined. In advance of determining of the percentages of Amp^R^ and Rif^R^, cells were transferred to fresh media and incubated for 24 h to differentiate whether temporary adaptive resistance occurred or not. Incubated cells were diluted and spotted or spread on Amp- or Rif-containing LB plates. **(B)** Amp susceptibility test of cells taken from each region (indicated by circles a, b, and c in **Figure 3A**) The same number of cells was spotted on plates containing Amp (0, 100, 150, and 200 μg/ml) and incubated for 24 h. Percentage of Amp-resistant (Amp^R^) **(C)** and Rif-resistant (Rif^R^) **(D)** cells taken from each region (indicated by circles a, b, and c in **Figure 3A**) Appropriate dilutions of the cells were spread on LB plates containing 50 μg/ml Rif or 200 μg/ml Amp. The gradation bar below the graph **(C,D)** indicates the estimated relative indole concentration in regions A, B, and C. All data represent the average of three replicates, and the error bar indicates the standard deviation.

Indole is a bacterially produced volatile compound (Kai et al., 2009; Kim and Park, 2013). In order to determine whether volatile indoles can give rise to AMR, we designed experiments in which volatile indole could be supplied from a distance (**Figure 3**). The indole emission point was separated from the agar media, but located within the upper left corner of a square petri dish (**Figure 3A**). Indole was slowly released from a paper disk containing 25 μ moles indole, and the same number of *P. putida* cells (10^5^ CFU per spot) was inoculated at constant intervals. After 24 h of incubation, results showed that cells located closer to the indole-emitting point grew well, even in the presence of Amp at a concentration that prevented the growth of *P. putida* cells on a control plate (**Figure 3A**, Plate C). Cells distant from the indole source could also survive, but they had irregular shapes and generated colonies of various sizes (**Figure 3A**, Plate C). This suggested that heterogeneity might be associated with indole concentration gradients and that cells grown far from the indole source might show phenotypic variation. To verify whether indole presence would cause mutational resistance or temporary adaptive resistance, cells were sampled from each region, transferred to fresh media, and incubated for 24 h before determining of mutated cells. Distant cells survived at high concentrations of Amp (**Figure 3B**) and showed high percentages of mutated cells compared to cells grown close to the indole source (**Figures 3C,D**). We verified that cells grown in single spots showed differences in temporal adaptation or mutations depending on the distance from the indole source. Thus, we speculated that cells could develop AMR by mutagenesis in the presence of low indole concentrations through spontaneously formed concentration gradients resulting from the volatility of indole.

### Heterogeneous Responses to Indole Enable the Induction of AMR in *P. putida* in Liquid Media

**FIGURE 4:**
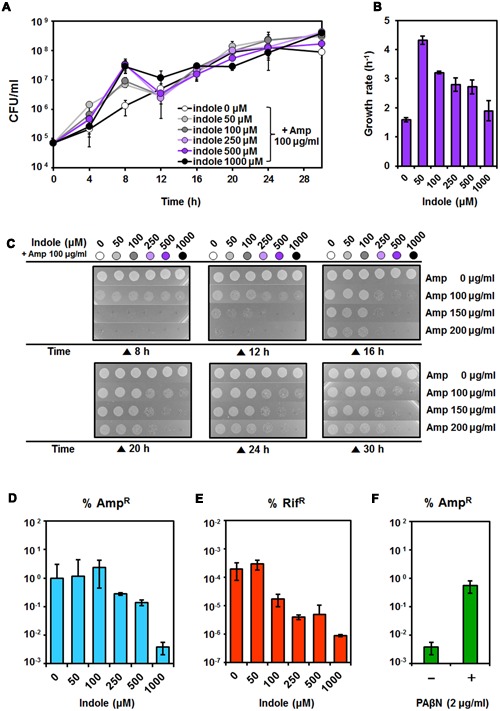
**Amp resistance induced by heterogeneous response to indole. (A)** Growth was monitored by counting colony forming units (CFUs) in the presence of indole with 100 μg/ml Amp. **(B)** Growth rates in each condition at 0–4 h of incubation, as shown in **Figure 4A**. **(C)** Amp susceptibility test of cells taken from cultures in 100 μg/ml Amp with various concentrations of indole, as shown in **Figure 4A**. Cells were harvested at the indicated time points (8, 12, 16, 20, 24, and 30 h of incubation) and washed to remove residual chemicals. The same number of cells was spotted on plates containing Amp (0, 100, 150, and 200 μg/ml) and incubated for 24 h. The percentage of mutated cells **(D,E)** was determined as follows. Cells harvested from cultures grown in 100 μg/ml Amp with various concentration of indole, as shown in **Figure 4A**. The percentages of mutated cells showing Amp resistance (Amp^R^) **(D)** and Rif resistance (Rif^R^) **(E)** were determined from the relative percentages of CFU/ml [(CFU obtained from the antibiotic plate/total number of CFU obtained from the LB plate) × 100]. **(F)** Percentage of Amp-resistant (Amp^R^) cells in the presence of an efflux pump inhibitor. Cells were inoculated into LB medium containing 100 μg/ml Amp and 1000 μM indole with or without 2 μg/ml PAβN and incubated for 30 h at 30°C. Appropriate dilutions of the cells were spread on LB plates containing 200 μg/ml Amp. The total number of CFUs was determined on LB agar plates. All data represent the average of three replicates, and the error bar indicates the standard deviation.

Different properties of the cells acquiring Amp resistance in response to volatile indole during plate growth led us to monitor the growth patterns of *P. putida* KT2440 in various concentrations of indole (0, 50, 100, 250, 500, and 1000 μM) with Amp (0, 50, 75, 100, 150, and 200 μg/ml) in microtiter plates (Supplementary Figure S8). No apparent indole toxicity was observed at ~500 μM without Amp, as shown in our previous report (Kim et al., 2013), and cells failed to grow in 200 μg/ml Amp (data not shown). At 50–1000 μM indole, in addition to Amp, the initial growth rates were greater than that in Amp-only conditions, but cultures started to collapse at 10–15 h (Supplementary Figure S8). Growth was scaled up and monitored by counting CFUs in the presence of indole with 100 μg/ml Amp (**Figure 4A**). Growth curves based on CFUs of cells grown in indole also contained the part of sticking out from the normal curve during 4–12 h (**Figure 4A**). This suggested that some cells died, but that surviving cells continued to grow, and after 30 h, the cell densities reached in each condition were nearly identical (**Figure 4A**). This two-stage growth curve indicated that rapid adaptation or mutation induced by both compounds allowed cultures of *P. putida* to withstand Amp. In addition, initial growth rates increased with the addition of indole (**Figure 4B**). At the initial growth stage, cells showed improved growth in the presence of a low rather than a high concentration of indole, which inhibited growth and caused cellular toxic effects in cells by increasing the NADH/NAD^+^ ratio and reducing the ATP concentration because of perturbations in the membrane potential when indole crossed the membrane (Kim et al., 2013). Small amounts of indole can be rapidly consumed with contributing to cell growth, because many non-indole-producing bacteria degrade millimolar concentrations of indole within hours or 10s of hours (Lee et al., 2009, 2015a; Barret et al., 2011; Hachani et al., 2011). Thus, we hypothesized that heterogeneous responses might be induced by indole degradation when cells are exposed to antibiotics.

Heterogeneity was assessed by a susceptibility test (**Figure 4C**) of cells sampled at each time point from agar plates containing various concentrations of Amp and percentages of mutated cells with Amp and rifampicin (Rif) resistance (**Figures 4D,E**). To verify whether adaptive transitional resistance occurred or not, cells were transferred to fresh media and incubated for 24 h before determining of the percentage of mutated cells. We confirmed that cells grown in the absence of indole or presence of low concentrations (0, 50, or 100 μM) of indole could become more resistant to Amp because they were able to grow on 200 μg/ml Amp-containing plates (**Figure 4C**). However, cells incubated in high concentrations of indole (500 and 1000 μM) with Amp failed to grow in 200 μg/ml Amp-containing plates (**Figure 4C**), even though the number of them were similar with cells grown in other conditions after 30 h of incubation (**Figure 4A**). We also determined that the percentage of mutated cells decreased as the concentration of indole increased (**Figures 4D,E**). There was a small or no significant difference in mutated cells grown in the absence and or presence of low concentrations of indole, indicating that the effects of indole in these conditions might be attenuated by metabolism of indole and that mutagenesis induced by Amp could facilitate resistance. Thus, populations grown in the absence of indole or at low concentrations of indole showed more mutated cells (**Figures 4C–E**). However, the low percentage of mutated cells might be caused by antibiotics being pumped from the inside of cells, which could be essential for the acquisition of resistance, along with the generation of mutations in the presence of high concentrations of indole (~1000 μM) (**Figures 4C–E**), as confirmed in **Figure 2**. The percentage of mutated cells in high concentrations of indole (1000 μM) increased 1000 fold in the presence of the RND-type efflux pumps inhibitor PAβN (**Figure 4F**), suggesting that RND-type efflux pumps, rather than mutagenesis, were the main contributors to resistance when indole concentrations were sufficient to upregulate RND-type efflux pumps. Therefore, indole might induce heterogeneous responses in cells under antibiotic stress.

### Gene Expression Involving AMR Can Be Altered in the Presence of Indole

**FIGURE 5:**
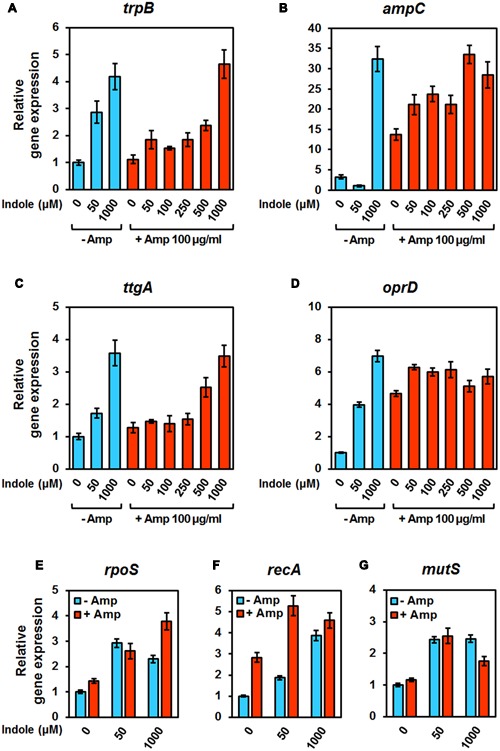
**Monitoring the expression of genes in response to indole concentrations in *P. putida* KT2440.** The expression of genes involved in **(A)** the tryptophan pathway (*trpB*), **(B–D)** Amp resistance (*ampC*, *ttgA*, and *oprD*) and **(E–G)** DNA repair/mutagenesis (*rpoS*, *recA*, and *mutS*) were evaluated. Total RNA was isolated from cultures after 8 h of incubation with 100 μg/ml Amp and indole (0, 50, 100, 250, 500, and 1000 μM) or indole alone (0, 50, and 1000 μM) without Amp. All data represent the average of three replicates, and the error bar indicates the standard deviation.

To identify paths to resistance against Amp, the expression of genes implicated in the tryptophan pathway (*trpB*), Amp resistance (*ampC*, *ttgA*, and *oprD*), and DNA repair/mutagenesis (*rpoS*, *recA*, and *mutS*) was verified under various concentrations of indole in the presence of Amp (**Figure 5**). The expression of *trpB* showed that the tryptophan pathway was induced by indole, regardless of the addition of Amp (**Figure 5A**). Two genes involved in Amp resistance, *ampC* (encoding a β-lactamase) and *ttgA* (encoding an RND transporter), were expressed highly and in an indole-concentration dependent manner (**Figures 5B,C**). Expression of *oprD*, which encodes the porin protein (Quale et al., 2006; Kumita et al., 2009), was increased in all treatment conditions relative to the control; however, there were no significant difference based on the presence of indole, Amp, and both (**Figure 5D**). The high levels of *oprD* expression encoding a porin that facilitates β-lactam antibiotics entrance into the cell (Thomson and Bonomo, 2005) implied that the effects of Amp could be similar in all conditions, even if the concentrations of indole varied. Thus, porin reduction was unlikely to contribute to the heterogeneity of cells in response to indole. It was speculated that indole might affect mutation evaluated by the expression of *rpoS* and *mutS*, which engages in bacterial mutagenesis in the presence of β-lactam antibiotics (Gutierrez et al., 2013), under indole treatments regardless of the concentration (**Figures 5E,G**). *recA* gene expression was increased over five fold in the presence of low concentrations of indole, suggesting that DNA repair may be necessary in these conditions (**Figure 5F**). Our gene expression analysis demonstrated that indole reduces Amp-induced mutagenesis by promoting the expression of TtgAB efflux pumps and an indole-induced β-lactamase in *P. putida* KT2440, and that indole alone does not alter the expression of many mutagenesis-related genes such as *rpoS*, *recA*, and *mutS*.

## Discussion

Indole has received considerable attention because of its broad range of effects on various microbial functions (Kim and Park, 2015). Indole increases AMR through the expression of transporter and stress resistance genes and the formation of bacterial persister cells (Hirakawa et al., 2005; Vega et al., 2012, 2013; Molina-Santiago et al., 2014). Here, we first report that aerial exposure to the volatile compound indole can increase AMR of in bacteria distant from the indole source by modulating antibiotic-induced heterogeneity and increasing the expression of RNA-type efflux pumps and the activity of β-lactamase in *P. putida* KT2440. Expression, enzyme activities, and mutational analyses indicated that AmpC β-lactamase is important for acquiring Amp resistance of *P. putida* in the presence of indole. To better differentiate the AmpC β-lactamase and RND-type efflux pump contribution to Amp resistance, we used carbenicillin (Car), which is one of the β-lactam classes antibiotics and much more stable than ampicillin, for determining MICs along with Amp in the *ΔampC* cells. MIC of Amp (256 μg/ml in wild type; 64 μg/ml in *ΔampC*) was lowered, but MIC of Car (512 μg/ml in both strains) did not change in the *ΔampC* mutant compared to wild type, indicating that this AmpC β-lactamase might be contributing more for acquiring indole-induced Amp resistance. Recently, it was reported that direct recognition of β-lactam antibiotics by a histidine kinase receptor may induces resistance mechanism in *E. coli* (Li et al., 2016). Further studies are needed to reveal molecular mechanisms involved in indole-induced Amp resistance in *P. putida*.

Non-indole-producing *P. aeruginosa* can completely metabolize 0.5 mM of indole within 10 h (Lee et al., 2009), and degradation of indole has also been verified in other bacteria such as *Arthrobacter*, *Alcaligenes*, and *Agrobacterium* (Arora and Bae, 2014; Lee et al., 2015a; Kim et al., 2016). In our transcriptome analysis, several oxygenase-encoding genes and genes involved in tryptophan metabolism were upregulated in response to indole (Supplementary Figure S4, Supplementary Table S4), suggesting that indole was quickly metabolized by *P. putida* and that it enabled cells to grow in the presence of Amp by promoting growth initially (**Figure 4B**). Thus, small amounts of indole that affect initial growth could diminish with continued growth, and then Amp-induced mutagenesis might contribute to the acquisition of Amp resistance. This could explain that final population grown at low concentration of indole plus Amp showed high percentages of mutated cells (**Figures 3**, **4**). High concentrations of indole might degrade slowly, thus allowing the remaining indole to upregulate genes involved in AMR, as shown in our gene expression study, which reduced Amp-induced heterogeneity. In this study, we examined bacterial heterogeneity via susceptibility testing and determining the percentages of cells resistant to Amp or Rif in culture. These resistant cells might be not temporally adapted, but mutated instead, because the cells had passed through dozens of generations during the long incubation. Further studies are needed to determine which genes are mutated and whether these mutations are heritable.

The expression of multidrug transporters is enhanced by indole in *E. coli* (Hirakawa et al., 2005; Nikaido et al., 2012), *Salmonella* (Vega et al., 2012), *Agrobacterium* (Lee et al., 2015a) and *Pseudomonas* (Molina-Santiago et al., 2014). Recently, the plasmid-encoded TtgGHI efflux pump in cells exposed to indole were reported to play more important roles in acquiring AMR than those of the chromosomally located efflux pump TtgABC in *P. putida* DOT-T1E (Molina-Santiago et al., 2014). However, results of this study show that 12 genes involved in RND efflux pumps, including TtgABC, were upregulated over 1.5 fold in *P. putida* in response to indole, and that the absence of *oprM* and *mexB* genes had severe effects on the acquisition of Amp resistance in *P. aeruginosa* in response to indole treatment (Supplementary Table S6, **Figure 2C**). The decrease in survival under Amp with indole when RND-type efflux pumps inhibitor PAβN treated indicated that RND-type efflux pumps were essential for acquisition of AMR originated from the indole addition (**Figure 2A**). It will be necessary to examine the molecular mechanisms underlying indole regulation of efflux pump expression. Even in the absence of *oprM* or *mexB*, cells could be resistant to Amp with exposure to indole at concentrations less than that contributing to resistance in the wild-type strain (**Figure 2C**). In contrast, other Amp-sensitive strains required more indole to acquire resistance (Supplementary Figure S6), and indole contributes to Amp resistance, even if the bacteria already showed some resistance (Supplementary Figure S7). Thus, the effective concentrations of indole to drive AMR might differ by individual. The fact that the bacteria could acquire resistance to antibiotics with adjustments in the concentration of indole, even though characteristics of these bacteria, such as susceptibility to antibiotics, differed between them, and they could not upregulate efflux pumps or β-lactamases (**Figures 2C,D**), suggested that indole might contribute to AMR though various routes.

Based on many studies regarding AMR induced by indole, this physiological function was relevant with indole concentrations of 0.25–4.0 mM (Hirakawa et al., 2005; Nikaido et al., 2012; Vega et al., 2012; Molina-Santiago et al., 2014; Lee et al., 2015a). This concentration is similar to that in *E. coli* cultures in the stationary phase (Li and Young, 2013; Gaimster et al., 2014). We confirmed that 50 μM–1 mM indole could promote Amp resistance in this study (**Figure 4A**, Supplementary Figure S8), and that a low concentration of indole with long-term exposure could promote survival in the presence of Amp by supporting growth (**Figure 4B**). The fact that indole affected cell physiologies at a low concentration should not be overlooked, because indole is widespread in natural environments and is volatile. Millmolar concentrations of indole can be toxic and inhibit growth (Kim et al., 2013; Kim and Park, 2015; Lee et al., 2015a), protein folding (Kim and Park, 2013) and cell division, acting as a proton ionophore (Chimerel et al., 2012). Indole can increase the NADH/NAD^+^ ratio and decrease the ATP concentration in cells because of perturbations in the membrane potential when indole is transported across the cell membrane (Piñero-Fernandez et al., 2011; Chimerel et al., 2012; Kim et al., 2013). However, this toxicity might also accompany global effects on the expression of genes involved in stress defense, such as those that encode chaperones, proteases, and efflux pumps, which alleviate oxidative stress and the phage shock response (Supplementary Tables S3–S9). These effects could result in AMR, even if mutations were reduced by high concentration of indole (Hirakawa et al., 2005; Nikaido et al., 2012; Vega et al., 2012, 2013; Kim and Park, 2013; Kim et al., 2013). A small amount of indole was enough to promote growth initially (**Figure 4B**), although it might not be sufficient to activate the above mentioned resistance mechanisms by itself. The Rif^R^ mutation frequency was quite low (10^-7^~10^-6^) in the presence of indole alone without Amp treatment, and there were no significant difference in mutation rates with various indole concentrations (0–1000 μM). Thus, indole might fail to affect the mutation rate when supplied alone or boost the effects of Amp when cells are exposed to both indole and antibiotics. Low levels of indole might facilitate antibiotic-mediated mutagenesis with long exposure times, based on the promotion of growth followed by active bacterial metabolism.

β-lactam antibiotics can promote mutagenesis via an RpoS-regulated stress response (Gutierrez et al., 2013), perturbing the TCA cycle and increasing ROS production (Kohanski et al., 2007, 2010; Belenky et al., 2015). According to our gene expression analysis, genes involved in DNA damage repair (*recA*), mutagenesis, and oxidative stress defense were upregulated by indole in the presence of Amp, but not by indole alone (Supplementary Table S9). In addition, we found, in our previous (Kim et al., 2013) and current studies, that the expression of TCA cycle genes was increased by indole, regardless of the exposure time (Supplementary Table S3). Whether indole can cause oxidative stress is still unclear, although several genes implicated in oxidative stress defense were increased in indole and Amp mixed treatments (Supplementary Table S9). Those findings suggest that antibiotic-mediated mutagenesis might be promoted by indole and confer AMR.

Many bacteria produce secondary metabolites that have low molecular weights and high vapor pressure, and that can easily evaporate and diffuse through heterogeneous conditions that might promote adaptation in response to environmental changes (Bernier et al., 2011; Audrain et al., 2015). Until now, the impact of indole on drug resistance has been verified only with physical interactions between microorganisms and indole in culture media (Hirakawa et al., 2005; Lee H.H. et al., 2010; Nikaido et al., 2012; Vega et al., 2012, 2013). Here, we provide the first report that airborne indole affects AMR by promoting heterogeneous responses, depending on the distance from the indole source (**Figure 3A**). The indole derivate indole-3-acetic acid and tryptophan promote Amp resistance (Supplementary Figure S9), indicating that indole-like compounds can increase AMR when they pass through cellular membranes or are metabolized in intracellular spaces. AMR induced by indole was verified, irrespective of the indole-producing abilities of the tested bacteria, media types, and class of antibiotics, suggesting that the acquisition of resistance in response to long-term exposure to indole might be common (**Figure 1**, Supplementary Figures S2, S9). Therefore, further genetic and physiological studies, along with ecological research on indole in microbial communities, are necessary to clarify the role of indole at various concentrations in the acquisition of AMR.

## Author Contributions

JK and WP designed and coordinated the study. JK, BS, and CP performed the experiments and collected the data. JK wrote the first complete draft of the manuscript. WP provided substantial modifications. All authors contributed to and approved the final version of the manuscript.

## Conflict of Interest Statement

The authors declare that the research was conducted in the absence of any commercial or financial relationships that could be construed as a potential conflict of interest.
