# Supplementary material for: Indole-Induced Activities of β-Lactamase and Efflux Pump Confer Ampicillin Resistance in Pseudomonas putida KT2440
Source: Front Microbiol. 2017 Mar 14;8:433. doi: 10.3389/fmicb.2017.00433 (PMC5348495; doi:10.3389/fmicb.2017.00433)
Supplement: Supplementary file 1 [file Data_Sheet_1.docx]

**Supplementary Material**

**Indole-Induced Activities of β-Lactamase and Efflux Pump Confer Ampicillin Resistance in *Pseudomonas putida* KT2440**

Jisun Kim, Bora Shin, Chulwoo Park and Woojun Park^*^

Laboratory of Molecular Environmental Microbiology, Department of Environmental Sciences and Ecological Engineering, Korea University, Seoul, Republic of Korea

* Correspondence: Woojun Park, wpark@korea.ac.kr

**Supplementary Figure S1. Killing assay in the presence of indole in *P. putida* KT2440.** Cells were grown overnight in Luria-Bertani (LB) medium and subsequently diluted 100 fold. Exponentially growing cells were pre-incubated for 1 h with (blue) and without (gray) 1 mM indole and treated with 200 μg/ml ampicillin. Cells were treated with indole and ampicillin at the same time (red). The cells were sampled at the indicated times and washed two times. Then, cells were diluted in phosphate-buffered saline and spread onto LB plates. Colonies were counted after an overnight incubation at 30 °C. All data are presented as the average of three replicates, and error bars indicate the standard deviation.

**
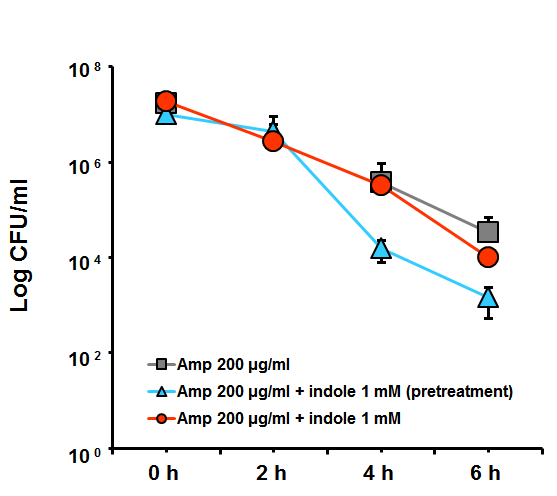
**

**Supplementary Figure S2. Antibiotic resistance induced by indole in *Acinetobacter oleivorans* DR1 and *Escherichia coli* O157:H7. (A)** Ampicillin (Amp) susceptibility test with or without 1 mM indole in *A. oleivorans* DR1 and *E. coli* O157:H7. **(B)** Tetracycline (Tet) susceptibility test with or without 1 mM indole in *A. oleivorans* DR1. The exponentially growing cells were harvested and washed twice with phosphate-buffered saline (PBS). Approximately 10^7^ CFU/ml cells were inoculated into PBS and serially diluted. Each dilution of the solution was spotted on plates and incubated for 24 h at the optimal temperature for the strain.

**
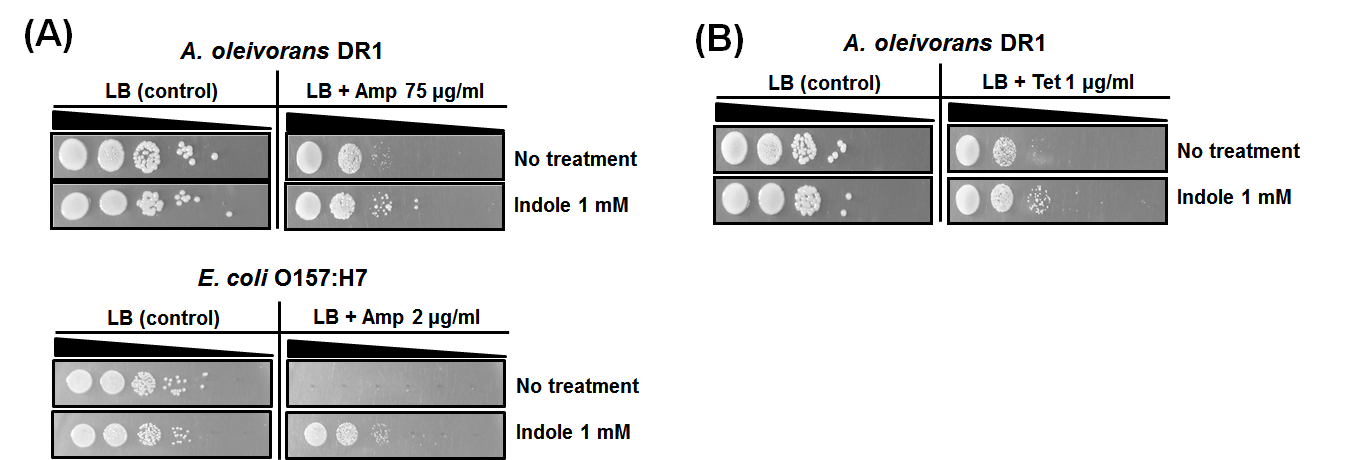
**

**Supplementary Figure S3. A schematic of the method of cell preparation for microarray.** *P. putida* KT2440 cells were grown overnight in Luria-Bertani (LB) medium and diluted 100 fold. When the diluted cells reached the exponential phase (OD_600_ ~0.4), cells were collected and washed twice with phosphate-buffered saline. Appropriate dilutions of the cells were spread on LB plates containing 1 mM indole, 50 μg/ml ampicillin, or both 1 mM indole and 50 μg/ml ampicillin. After 12 h of incubation at 30 °C, cells were collected from the plates, which contained approximately 100 colonies per plate, and total RNA was isolated.

**
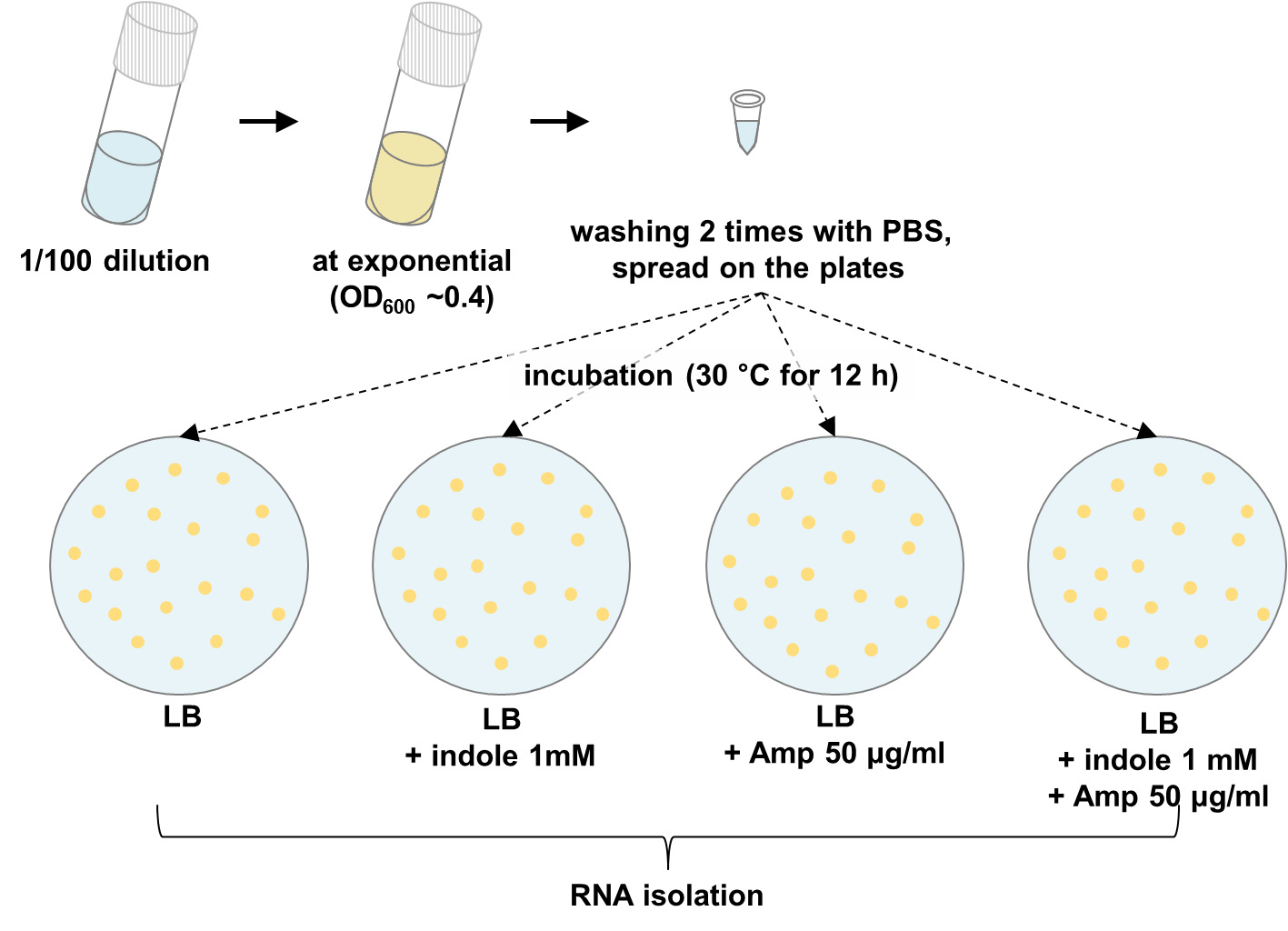
**

**Supplementary Figure S4. Expression of genes involved in the tryptophan metabolic pathway in the presence of indole.** Compounds participating in the pathway are represented by black dots beside their names. Gene symbols and locus tags of genetic elements responsible for each process are presented. The colors of the bars connecting two compounds indicate the gene expression level determined by microarray.

**
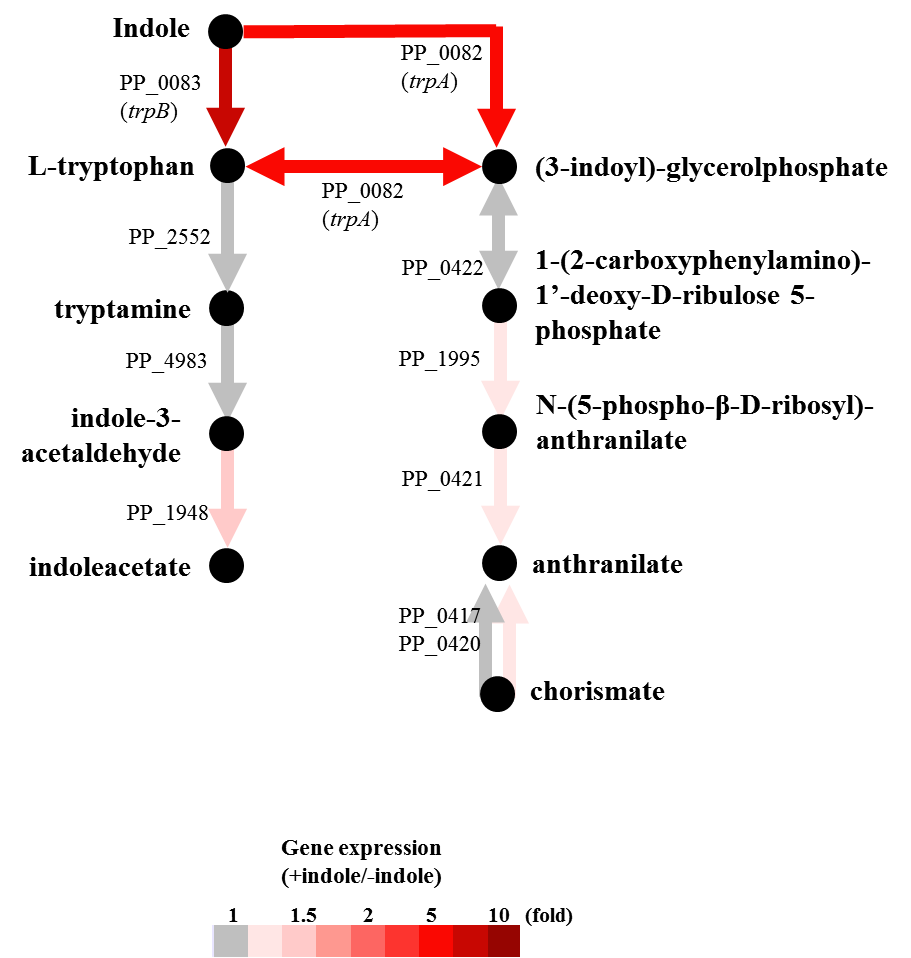
**

**Supplementary Figure S5. Susceptibility to ampicillin of various *P. aeruginosa* mutant strains in the presence or absence of indole.** Each strain had a mutation in a gene highly expressed under both ampicillin (Amp) and indole, based on our transcriptome analysis. The list of strains is presented in Supplementary Table 7. Exponentially growing cells were harvested by centrifugation and washed twice with phosphate-buffered saline. Cells were serially diluted and then spotted on a Luria-Bertani (LB) agar plate. The susceptibilities to ampicillin of some strains, which showed different sensitivities to ampicillin (indicated by red or blue), were confirmed in Supplementary Fig. S6 and Supplementary Fig. S7.

**
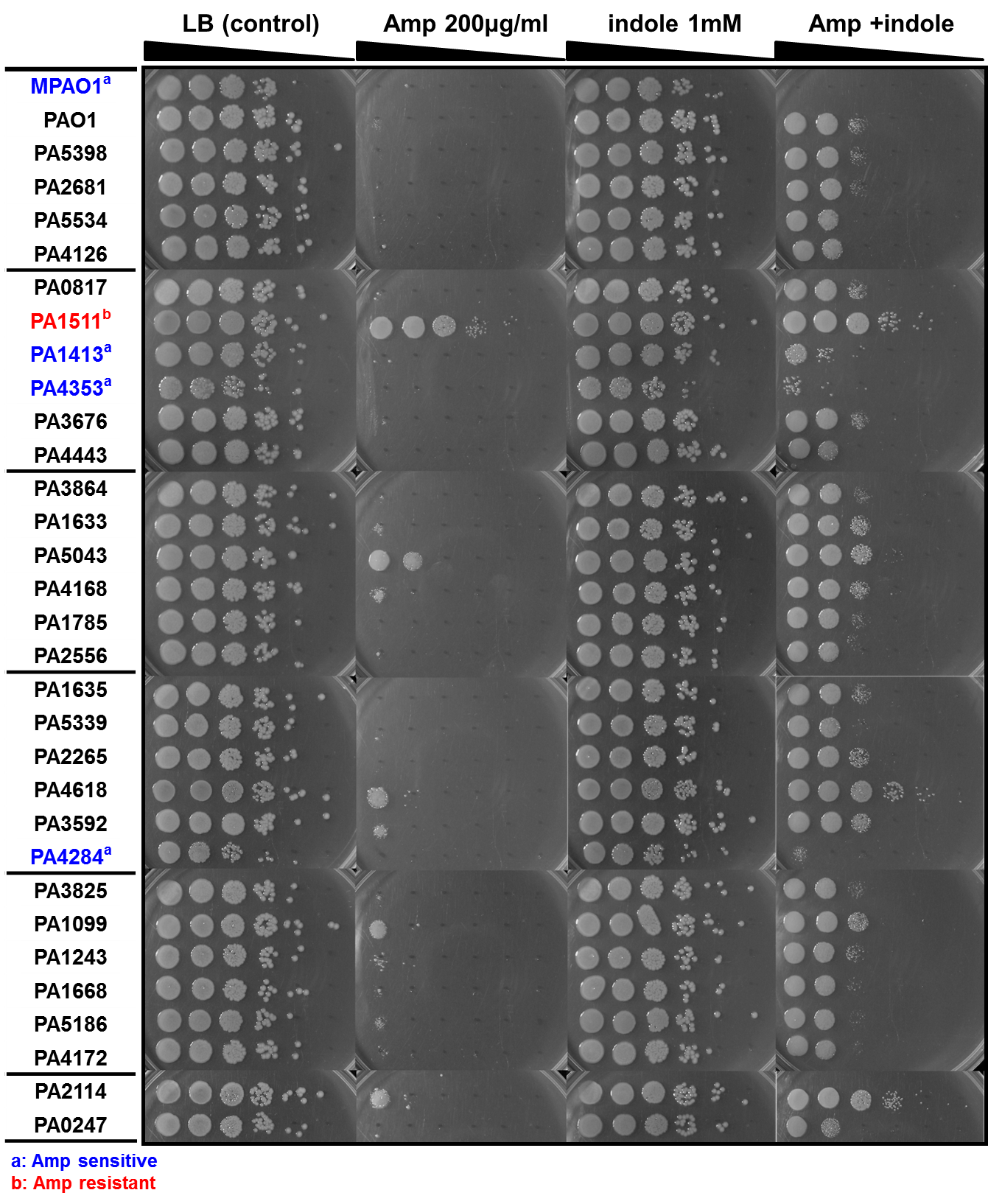
**

**Supplementary Figure S6. Susceptibility to ampicillin of ampicillin-sensitive strains in the presence or absence of indole.** *P. aeruginosa* MPAO1, PA1413 (encoding the LysR family transcriptional regulator), PA4353, and PA4284 (encoding exodeoxyribonuclease V, beta subunit) mutants showed greater ampicillin sensitivity than that of the other strains tested. Their susceptibilities to ampicillin in the presence of indole were confirmed by altering the concentrations of indole and ampicillin.


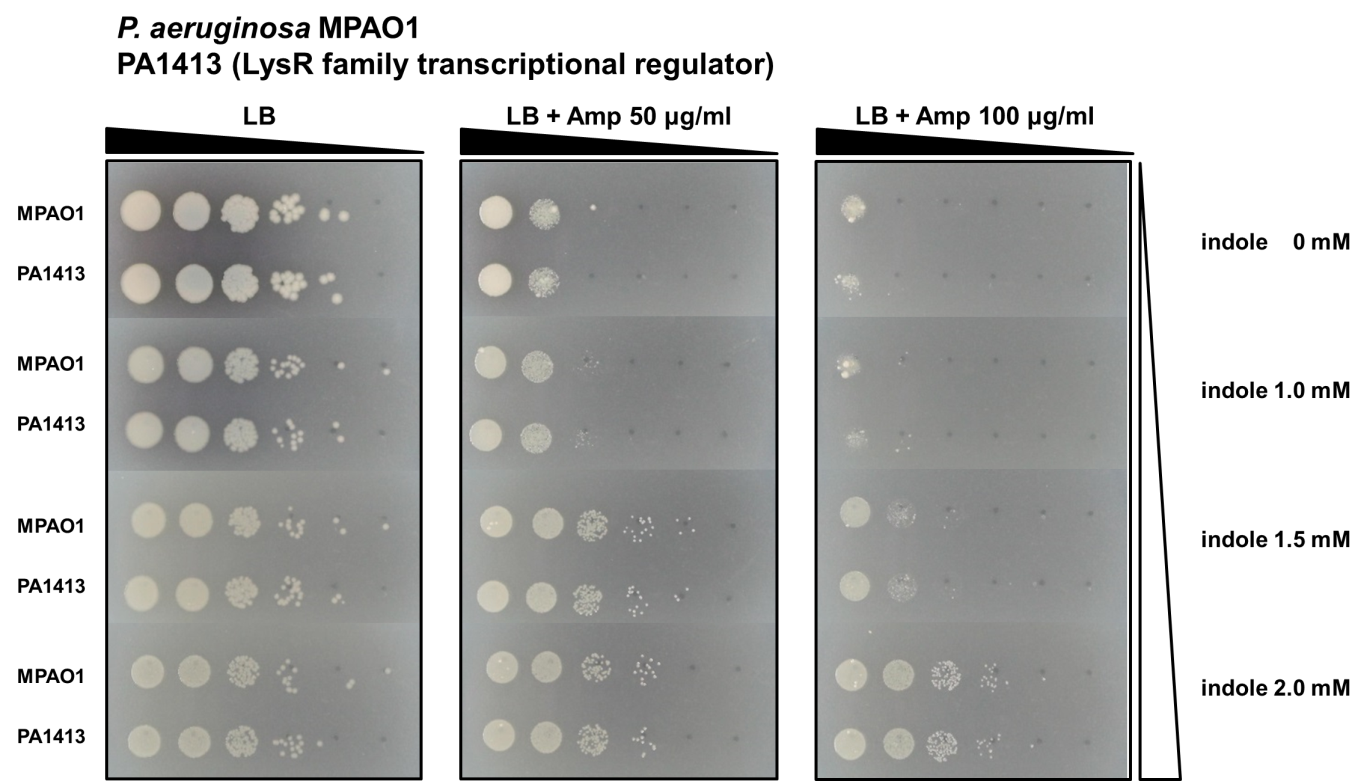


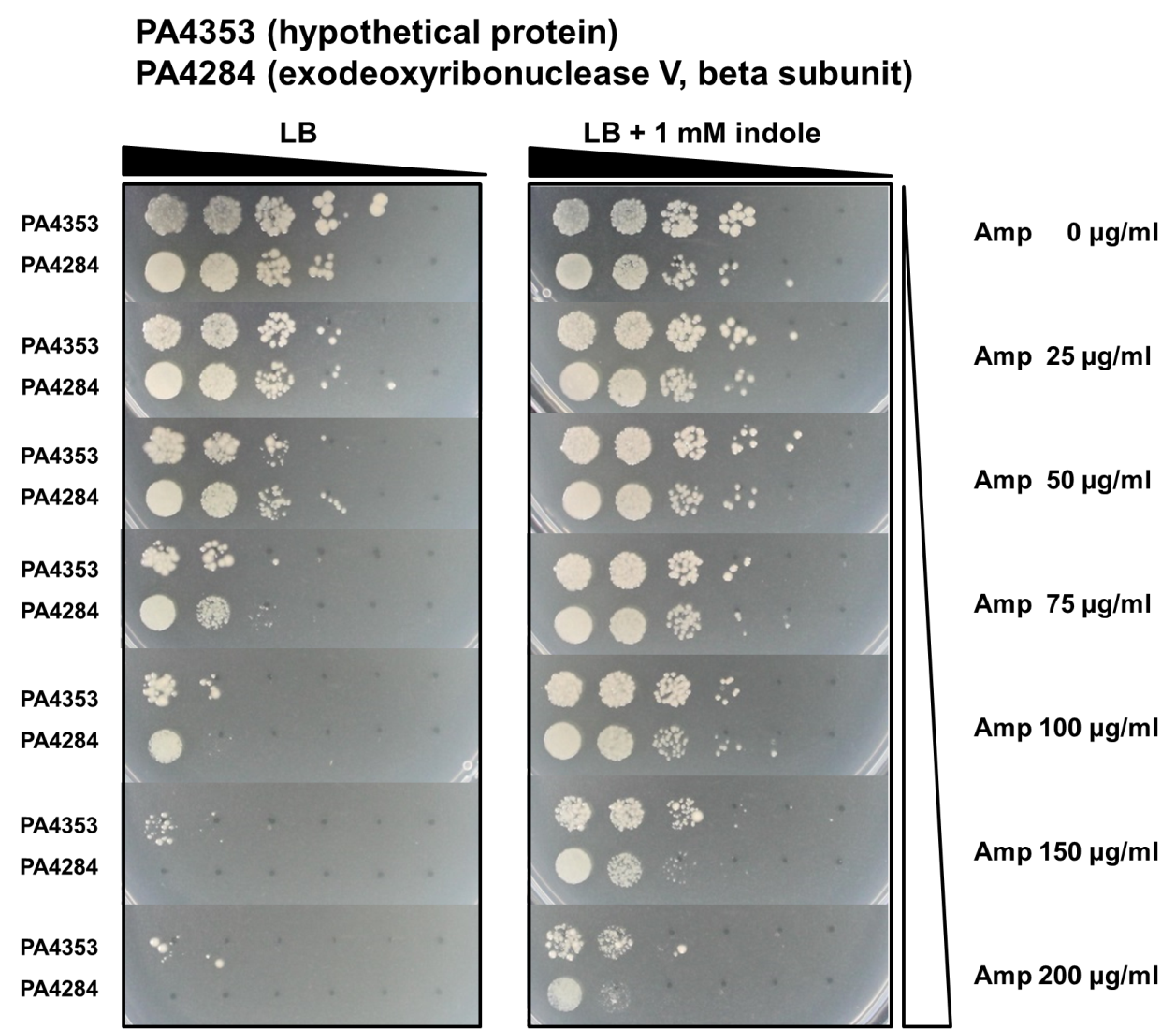


**Supplementary Figure S7. Susceptibility to ampicillin of ampicillin-resistant strains in the presence or absence of indole.** *P. aeruginosa* PA1511 (encoding the VgrG protein) mutants showed less ampicillin sensitivity than that of other strains tested. Their susceptibilities to ampicillin in the presence of indole were confirmed by altering the concentrations of ampicillin.


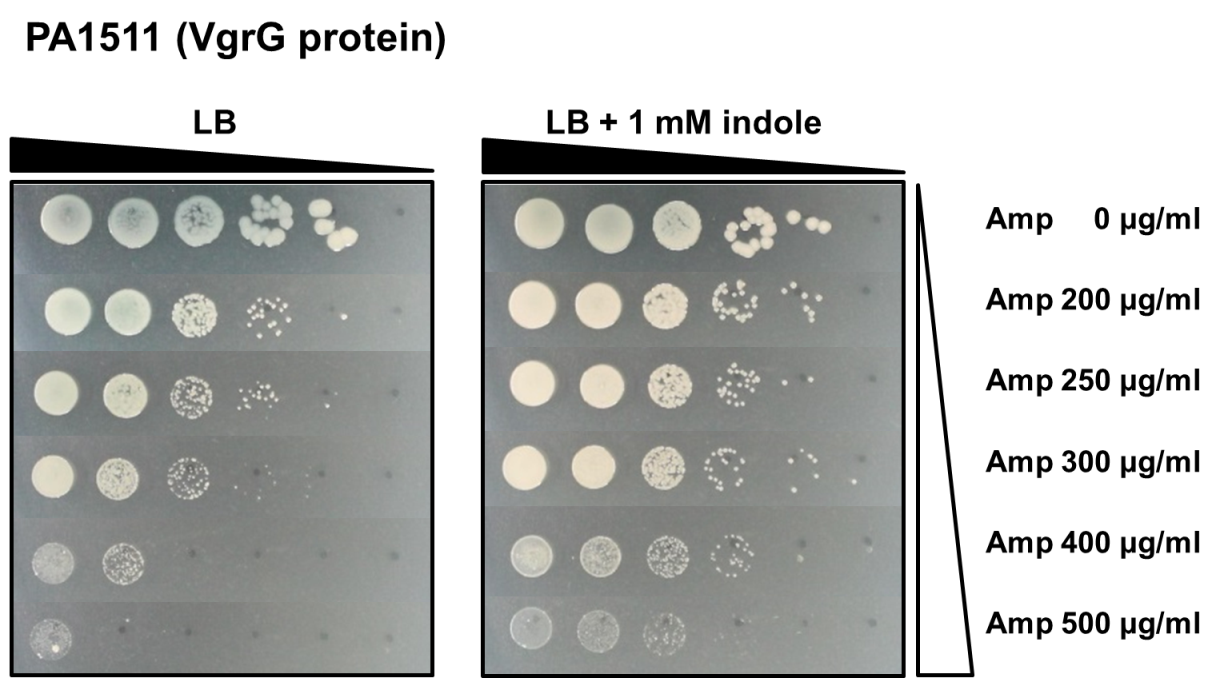


**Supplementary Figure S8. Growth patterns of *P. putida* KT2440 in various concentrations of indole and ampicillin.** Approximately 10^6^ CFU/ml cells were inoculated into Luria-Bertani (LB) medium supplemented with various concentrations of indole (0, 50, 100, 250, 500, or 1000 μM) and ampicillin (0, 50, 75, 100, 150, or 200 μg/ml). Measurement of the OD_600_ was performed in microtiter plates, and cells were incubated at 30 °C. The last bar graph shows CFU/ml in each condition at 36 h of incubation with 150 μg/ml ampicillin. After 36 h of incubation, cells were collected, washed, diluted, and spread on LB plates. The bar graph indicates that the CFU/ml was slightly increased with the addition of indole.


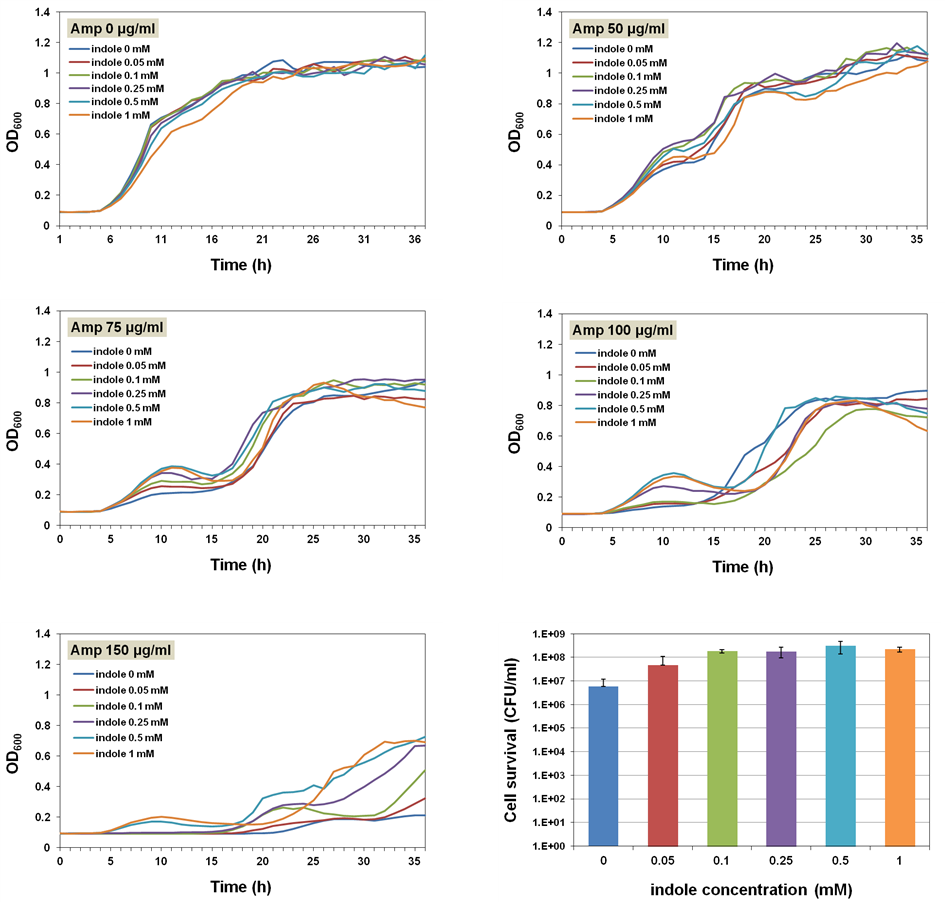


**Supplementary Figure S9. Acquisition of antibiotic resistance by *P. putida* in response to indole-3-acetic acid and tryptophan.** Exponentially growing cells were harvested and washed twice with phosphate-buffered saline (PBS). Approximately 10^7^ CFU/ml cells were inoculated into PBS and serially diluted. Each dilution of the solution was spotted on plates and incubated at the optimal temperature for the strain for 24 h.

**
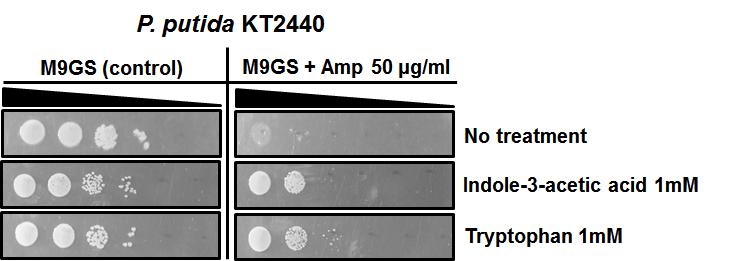
**

**Supplementary Table S1.** Bacterial strains, plasmids and primers used in this study.

| **Bacteria/ primers** | **Description** | **Source /** |
| --- | --- | --- |
|  |  | **Reference** |
| **Strains** |  |  |
| *Pseudomonas putida* KT2440 | TOL plasmid-cured derivative of *P. putida* mt-2 | Lab stock, 2 |
| KT2440R | Rifampicin-resistance strain of *P. putida* KT2440 | Lab stock, 3 |
| the *ampC* mutant | Insertion of pVIK112-*ampC* in *P. putida* KT2440R | This study |
| *Pseudomonas aeruginosa* |  |  |
| PAO1 | *P. aeruginosa* PAO1 wild type | Lab stock |
| MPAO1 | *P. aeruginosa* MPAO1 wild type |  |
| PA5398 | PA5398 (oxidoreductase, FMN-binding) mutant | All mutants from Washington  University Genome Center |
| PA2681 | PA2681(transcriptional regulator) mutant |  |
| PA5534 | PA5534 mutant |  |
| PA4126 | PA4126 (major facilitator family transporter) mutant |  |
| PA0817 | (ring-cleaving dioxygenase, putative) mutant |  |
| PA1511 | (vgrG protein) mutant |  |
| PA1413 | (transcriptional regulator, LysR family) mutant |  |
| PA4353 | PA4353 mutant |  |
| PA3676 | (RND efflux transporter) mutant |  |
| PA4443 | (sulfate adenylyltransferase, subunit 2) mutant |  |
| PA3864 | PA3864 mutant |  |
| PA1633 | PA1633 (potassium-transporting ATPase, A subunit) mutant |  |
| PA5043 | PA5043 (type IV pili biogenesis protein PilN) mutant |  |
| PA4168 | PA4168 (outer membrane ferric siderophore receptor) mutant |  |
| PA1785 | PA1785 (response regulator NasT) mutant |  |
| PA2556 | PA2556 (transcriptional regulator, AraC family) mutant |  |
| PA1635 | PA1635 (potassium-transporting ATPase, C subunit) mutant |  |
| PA5339 | PA5339 (endoribonuclease, putative) mutant |  |
| PA2265 | PA2265 (gluconate dehydrogenase, putative) mutant |  |
| PA4618 | PA4618 (xanthine dehydrogenase accessory factor) mutant |  |
| PA3592 | PA3592 (CAIB/BAIF family protein) mutant |  |
| PA4284 | PA4284 (exodeoxyribonuclease V, beta subunit) mutant |  |
| PA3825 | PA3825 mutant |  |
| PA1099 | PA1099 (response regulator FleR) mutant |  |
| PA1243 | PA1243 (response regulator) mutant |  |
| PA1668 | PA1668 mutant |  |
| PA5186 | PA5186 (alcohol dehydrogenase, iron-containing) mutant |  |
| PA4172 | PA4172 (exodeoxyribonuclease III, putative) mutant |  |
| PA2114 | PA2114 (major facilitator family transporter) mutant |  |
| PA0247 | PA0247 (4-hydroxybenzoate hydroxylase) mutant |  |
| PA4110 | PA4110 (beta-lactamase) mutant |  |
| PA0426 | PA0426 (multidrug/solvent RND transporter TtgB) mutant |  |
| PA0427 | PA0427 (multidrug/solvent RND outer membrane protein) mutant |  |
| *Acinetobacter oleivorans* DR1 | diesel oil degrader | Lab stock, 4 |
| *Escherichia coli* O157:H7 43894 | Shigatoxin 1, 2 producing *E. coli* O157:H7 | Lab stock |
| **Plasmids** |  |  |
| pVIK112 | R6K*oriV*, suicide vector, LacZ translation fusion vector; Km^R^ | Lab stock, 5 |
| pVIK112-*hslU* | Insertion of partial *hslU* regions in pVIK112 | This study |
| **Primers** | **Sequence 5′-3′** |  |
| KT *ampC* ex-F | CGACCAGATCATCCGCCCGCTCAT | |
| KT *ampC* ex-R | GGGTGGCCGTGTAGGTCTTGCTCA | |
| KT *trpB* ex-F | CAAGCGCATGGGCAAAAAG | |
| KT *trpB* ex-R | CCGGCGGTAACTGGCACGAT | |
| KT *ttgA* ex-F | AAGGAAGGCCAGCAGTTGTATCAG | |
| KT *ttgA* ex-R | TTTGGCATTGGCGTCGTCGTATTC | |
| KT *rpoS* ex-F | GAGCCCTCGCCGGAAGAAATC | |
| KT *rpoS* ex-R | GGTCGGGCGGTCGTCGGTCAG | |
| KT *recA* ex-F | GGCAGCCCGGAAACCACTACC | |
| KT *recA* ex-R | CTGACGGAACGGAGGCGAGACC | |
| KT *mutS* ex-F | CGCGTGCACGGCTACTTCAT | |
| KT *mutS* ex-R | CCAGGGCGCGGCTCTTG | |
| KT *oprD* ex-F | CGTGCTGCCCGTTGATAA | |
| KT *oprD* ex-R | TGGCAGCAGGCGAGAGT | |
| KT *ampC* SC-F | CGCGAATTCGCTGGTTTTGATGCCGTAGG | |
| KT *ampC* SC-F | CGCGGTACCCCGTACAAACACCGCAACAG | |

**Supplementary Table S2.** MICs (μg/ml) of several antibiotics for *P. putida* KT2440.

| **Liquid (LB)** | **MIC (μg/ml)** | **MIC with indole 1mM** |
| --- | --- | --- |
| Ampicillin | 256 | 256 |
| Kanamycin | 4 | 4 |
| Tetracycline | 4 | 4 |
| Chloramphenicol | 256 | 128 |
| Rifampicin | 16 | 16 |
| Norfloxacin | 4 | 4 |
| Gentamacin | 16 | 8 |
| Ticarcillin | 512 | 1024 |
| Carbenicillin | 1024 | 1024 |
| Zeocin | > 512 | > 512 |
| Apramycin | 16 | 16 |

**Supplementary Table S3.** Comparison of the gene expression levels in cells grown in liquid and agar plates in the presence of 1 mM indole. This list of differentially expressed genes with indole treatment in liquid growth was adapted from Kim et al*.* (2013).

| **Locus**  **_tag** | **Gene** | **Gene product** | **Liquid** | **Agar plate** | | |
| --- | --- | --- | --- | --- | --- | --- |
|  |  |  | **Indole**  **1 mM** | **Indole 1 mM** | **Amp**  **50 μg/ml** | **Indole**  **+Amp** |
| PP_0083 | *trpB* | tryptophan synthase, beta subunit | 3.52 | 7.56 | 0.91 | 8.00 |
| PP_5000 | *hslV* | heat shock protein HslV | 2.96 | 2.19 | 1.50 | 1.94 |
| PP_0154 |  | acetyl-CoA hydrolase/transferase family protein | 2.81 | 1.80 | 1.07 | 1.62 |
| PP_5001 | *hslU* | heat shock protein HslVU, ATPase subunit HslU | 2.75 | 1.97 | 1.03 | 1.71 |
| PP_4179 | *htpG* | heat shock protein HtpG | 2.62 | 2.33 | 1.16 | 1.98 |
| PP_4728 | *grpE* | heat shock protein GrpE | 2.56 | 1.99 | 1.81 | 2.09 |
| PP_4727 | *dnaK* | dnaK protein | 2.17 | 1.55 | 1.37 | 1.69 |
| PP_4188 | *kgdB* | 2-oxoglutarate dehydrogenase, dihydrolipoamide | 2.16 | 1.36 | 1.36 | 1.57 |
| PP_1982 | *ibpA* | heat-shock protein IbpA | 2.11 | 2.20 | 1.12 | 1.56 |
| PP_0625 | *clpB* | ATP-dependent Clp protease, ATP-binding subunit | 2.03 | 0.86 | 0.47 | 0.58 |
| PP_1157 |  | acetolactate synthase, catabolic, putative | 2.03 | 1.94 | 1.30 | 2.05 |
| PP_4012 |  | isocitrate dehydrogenase, NADP-dependent, | 2.03 | 0.98 | 1.26 | 1.07 |
| PP_0082 | *trpA* | tryptophan synthase, alpha subunit | 2.02 | 5.59 | 0.96 | 5.76 |
| PP_4189 | *kgdA* | 2-oxoglutarate dehydrogenase, E1 component | 2.00 | 1.31 | 1.48 | 1.85 |
| PP_2334 |  | carboxyvinyl-carboxyphosphonate | 1.93 | 1.40 | 1.06 | 1.47 |
| PP_1361 | *groEL* | chaperonin, 60 kDa | 1.92 | 2.13 | 1.30 | 1.77 |
| PP_3365 |  | acetolactate synthase, catabolic, putative | 1.90 | 1.75 | 1.15 | 1.94 |
| PP_2302 | *lon-2* | ATP-dependent protease La | 1.86 | 1.12 | 1.01 | 1.03 |
| PP_1443 | *lon-1* | ATP-dependent protease La | 1.83 | 1.80 | 1.21 | 1.06 |
| PP_2920 |  | conserved hypothetical protein | 1.80 | 1.32 | 0.54 | 1.30 |
| PP_4191 | *sdhA* | succinate dehydrogenase, flavoprotein subunit | 1.79 | 1.35 | 1.33 | 1.38 |
| PP_4194 | *gltA* | citrate synthase | 1.79 | 1.75 | 1.24 | 1.76 |
| PP_4186 | *sucC* | succinyl-CoA synthetase, beta subunit | 1.78 | 1.35 | 0.99 | 1.47 |
| PP_4894 |  | host factor-I protein | 1.77 | 1.29 | 1.11 | 1.19 |
| PP_1360 | *groES* | chaperonin, 10 kDa | 1.76 | 2.54 | 1.38 | 2.54 |
| PP_4116 | *aceA* | isocitrate lyase | 1.74 | 0.68 | 0.64 | 0.76 |
| PP_2339 | *acnB* | aconitate hydratase 2 | 1.73 | 1.29 | 1.56 | 1.41 |
| PP_4190 | *sdhB* | succinate dehydrogenase, iron-sulfur protein | 1.72 | 1.20 | 1.14 | 1.42 |
| PP_2335 |  | methylcitrate synthase, putative | 1.69 | 1.16 | 1.63 | 2.42 |
| PP_4192 | *sdhD* | succinate dehydrogenase, hydrophobic membrane | 1.68 | 1.25 | 1.15 | 1.36 |
| PP_0597 | *mmsA-1* | methylmalonate-semialdehyde dehydrogenase | 1.65 | 0.63 | 0.30 | 0.46 |
| PP_4185 | *sucD* | succinyl-CoA synthetase, alpha subunit | 1.65 | 1.19 | 1.22 | 1.43 |
| PP_2337 |  | conserved hypothetical protein | 1.62 | 0.89 | 0.72 | 0.95 |
| PP_2151 | *sthA* | soluble pyridine nucleotide transhydrogenase | 1.62 | 1.33 | 1.11 | 1.46 |
| PP_4892 | *hflK* | HflK protein | 1.62 | 1.47 | 1.00 | 1.54 |
| PP_4725 | *dapB* | dihydrodipicolinate reductase | 1.59 | 1.45 | 1.10 | 1.49 |
| PP_2298 |  | hypothetical protein | 1.59 | 2.07 | 1.42 | 1.84 |
| PP_4519 | *tolC* | agglutination protein | 1.58 | 0.80 | 1.01 | 0.84 |
| PP_0951 | *rpoX* | sigma54 modulation protein | 1.58 | 0.60 | 0.37 | 0.30 |
| PP_4187 | *lpdG* | 2-oxoglutarate dehydrogenase, lipoamide | 1.56 | 1.20 | 1.22 | 1.54 |
| PP_0897 |  | fumarate hydratase, class I | 1.50 | 1.68 | 0.96 | 1.65 |
| PP_4252 | *ccoQ-1* | cytochrome c oxidase, cbb3-type, CcoQ subunit | 0.67 | 0.54 | 0.31 | 0.39 |
| PP_0463 | *rpsQ* | ribosomal protein S17 | 0.67 | 0.82 | 1.27 | 0.97 |
| PP_1591 | *rpsB* | ribosomal protein S2 | 0.66 | 1.06 | 1.03 | 1.08 |
| PP_1772 | *rpsA* | ribosomal protein S1 | 0.66 | 0.94 | 0.96 | 0.99 |
| PP_4490 | *phhA* | phenylalanine-4-hydroxylase | 0.63 | 0.08 | 0.76 | 0.09 |
| PP_1185 | *oprH* | outer membrane protein H1 | 0.59 | 0.59 | 0.82 | 0.80 |

**Supplementary Table S4.** Oxygenase or cytochrome C oxidase genes expression in the presence of 1 mM indole, 50 μg/ml ampicillin, or both 1 mM indole and 50 μg/ml ampicillin.

| **Locus_tag** | **Gene product** | **Indole 1mM** | **Amp**  **50 μg/ml** | **Indole**  **+Amp** |
| --- | --- | --- | --- | --- |
| PP_3328 | ring-cleaving dioxygenase, putative | 20.263 | 2.062 | 13.115 |
| PP_4621 (*hmgA*) | homogentisate 1,2-dioxygenase | 0.958 | 2.235 | 2.970 |
| PP_0238 (*ssuD*) | organosulfonate monooxygenase | 0.056 | 1.689 | 2.160 |
| PP_2582 | heme oxygenase, putative | 1.046 | 1.244 | 2.047 |
| PP_2765 | sulfonate monooxygenase MsuD, putative | 1.644 | 1.022 | 1.363 |
| PP_1005 (*hemO*) | heme oxygenase | 1.051 | 0.795 | 1.314 |
| PP_3219 | alkansulfonate monooxygenase, putative | 1.246 | 0.831 | 1.312 |
| PP_3433 (*hpd*) | 4-hydroxyphenylpyruvate dioxygenase | 0.871 | 1.795 | 1.306 |
| PP_0383 | tryptophan 2-monooxygenase, putative | 0.656 | 2.947 | 1.264 |
| PP_3218 | monooxygenase, NtaA/SnaA/SoxA family | 1.494 | 0.922 | 1.050 |
| PP_4705 | ring-cleaving dioxygenase, putative | 2.342 | 2.196 | 1.030 |
| PP_4466 | dioxygenase, TauD/TfdA family | 1.113 | 1.393 | 0.929 |
| PP_4655 (*pcaG*) | protocatechuate 3,4-dioxygenase, alpha subunit | 1.671 | 0.919 | 0.911 |
| PP_3713 (*catA*) | catechol 1,2-dioxygenase | 1.263 | 1.392 | 0.700 |
| PP_2568 | ring-cleaving dioxygenase | 1.200 | 0.888 | 0.606 |
| PP_3199 | monooxygenase, putative | 1.276 | 1.955 | 0.444 |
| PP_0106 | cytochrome c oxidase, subunit III | 1.322 | 1.061 | 3.854 |
| PP_4256 (*ccoO*-2) | cytochrome c oxidase, cbb3-type, subunit II | 2.760 | 0.922 | 1.678 |
| PP_0815 (*cyoD*) | cytochrome o ubiquinol oxidase, protein CyoD | 1.181 | 1.117 | 1.348 |
| PP_4193 (*sdhC*) | succinate dehydrogenase, cytochrome b556 | 1.208 | 1.136 | 1.258 |
| PP_5378 | cytochrome c family protein | 1.280 | 0.982 | 1.203 |
| PP_0813 (*cyoB*) | cytochrome o ubiquinol oxidase, subunit I | 1.029 | 1.076 | 1.194 |
| PP_3183 | SCO1/SenC family protein/cytochrome c | 5.148 | 1.446 | 1.190 |
| PP_2675 | cytochrome c-type protein | 1.991 | 0.988 | 1.069 |
| PP_1841 | cytochrome c family protein | 1.982 | 0.540 | 0.901 |

**Supplementary Table S5.** Beta-lactam resistance gene expression profiles in the presence of 1 mM indole, 50 μg/ml ampicillin, or both 1 mM indole and 50 μg/ml ampicillin.

| **Locus_tag** | **Gene product** | **Indole 1mM** | **Amp**  **50 μg/ml** | **Indole**  **+Amp** |  |
| --- | --- | --- | --- | --- | --- |
| **Loss or severe reduction of porins** | | | | | |
| PP_1206 (*oprD*) | porin D | 0.838 | 0.627 | 0.713 |  |
| **Beta-lactamase (class C)** | | | | | |
| PP_2876 (*ampC*) | beta-lactamase | 1.044 | 0.962 | 0.969 |  |
| PP_1239 | metallo-beta-lactamase family protein | 1.176 | 1.291 | 1.583 |  |
| PP_1775 | metallo-beta-lactamase family protein | 1.08 | 1.003 | 0.977 |  |
| PP_1952 | metallo-beta-lactamase family protein | 1.15 | 0.891 | 0.904 |  |
| PP_2045 | metallo-beta-lactamase family protein | 1.438 | 1.21 | 1.348 |  |
| PP_3291 | metallo-beta-lactamase family protein | 0.607 | 0.433 | 0.41 |  |
| PP_4033 | metallo-beta-lactamase family protein | 1.212 | 0.986 | 1.07 |  |
| PP_0052 | metallo-beta-lactamase family protein | 0.211 | 0.141 | 0.183 |  |
| PP_0772 | metallo-beta-lactamase family protein | 1.313 | 1.1 | 0.847 |  |
| **Inhibition of peptidoglycan biosynthesis, increase in muropeptides** | | | | | |
| PP_1355 | muropeptide permease AmpG | 1.089 | 0.996 | 0.971 |  |
| PP_2145 | beta-hexosaminidase | 1.001 | 1.412 | 1.31 |  |
| **Penicillin binding proteins** | | | | | |
| PP_5084 | penicillin-binding protein | 1.289 | 1.792 | 1.776 |  |
| PP_3741 (*mrdA-1*) | penicillin-binding protein 2 | 0.782 | 0.641 | 0.799 |  |
| PP_4807 (*mrdA-2*) | penicillin-binding protein 2 | 1.075 | 0.906 | 1.297 |  |
| PP_1331 (*ftsI*) | penicillin-binding protein 3 | 1.173 | 1.165 | 1.159 |  |
| PP_0572 (*pbpC*) | penicillin-binding protein 1C | 0.857 | 1.074 | 1.258 |  |
| PP_1331 | penicillin-binding protein 3 | 1.173 | 1.165 | 1.159 |  |
| PP_4683 (*mrcB*) | penicillin-binding protein | 1.32 | 1.094 | 1.206 |  |
| PP_1108 | acylase, penicillin amidase family | 0.966 | 1.036 | 0.789 |  |
| PP_2901 | penicillin amidase family protein | 1.033 | 0.984 | 0.829 |  |
| **RND efflux pumps** | | | | | |
| PP_1384 (*ttgC*) | multidrug/solvent RND outer membrane protein | 1.648 | 0.959 | 1.78 |  |
| PP_1798 | outer membrane efflux protein | 1.187 | 1.142 | 1.386 |  |
| PP_4923 | outer membrane efflux protein | 1.08 | 0.515 | 0.96 |  |
| PP_0906 | multidrug efflux RND transporter, putative | 1.143 | 0.989 | 1.007 |  |
| PP_1385 (*ttgB*) | multidrug/solvent RND transporter TtgB | 1.684 | 0.993 | 1.968 |  |
| PP_3456 | multidrug efflux RND transporter | 0.907 | 1.413 | 0.787 |  |
| PP_0907 | RND efflux membrane fusion protein-related | 0.804 | 0.888 | 1.301 |  |
| PP_1386 (*ttgA*) | multidrug/solvent RND membrane fusion protein | 3.095 | 1.457 | 3.981 |  |
| PP_3455 | multidrug efflux RND membrane fusion protein | 0.551 | 0.8 | 0.463 |  |

**Supplementary Table S6.** RND efflux pump-encoding gene expression profiles in the presence of 1 mM indole, 50 μg/ml ampicillin, or both 1 mM indole and 50 μg/ml ampicillin.

| **No.** | **Annotation** | **RND efflux pump** | **Locus tag** | **Indole 1 mM** | **Amp**  **50 g/ml** | **Indole**  **+Amp** |
| --- | --- | --- | --- | --- | --- | --- |
| 1 | Acriflavine resistance protein | AcrB1 | PP_3456 | 0.907 | 1.413 | 0.787 |
|  | Efflux transporter, RND family, MFP subunit | AcrE1 | PP_3455 | 0.551 | 0.800 | 0.463 |
| 2 | Toluene efflux pump outer membrane protein | TtgC | PP_1384 | 1.648 | 0.959 | 1.780 |
|  | Toluene efflux pump membrane transporter | TtgB | PP_1385 | 1.684 | 0.993 | 1.968 |
|  | Toluene efflux pump periplasmic linker protein | TtgA | PP_1386 | 3.095 | 1.457 | 3.981 |
| 3 | Hypothetical protein | ZncC | PP_3800 | 1.310 | 0.905 | 1.132 |
|  | Periplasmic solute binding protein (zinc transport system) | ZncA | PP_3801 | 1.157 | 0.893 | 1.328 |
|  | ABC transporter related protein (zinc transport system) | ZncB | PP_3802 | 1.142 | 0.919 | 1.282 |
| 4 | RND efflux system outer membrane lipoprotein | MacC | PP_4211 | 1.350 | 0.674 | 2.822 |
|  | Macrolide export ATP-binding/permease protein | MacA | PP_4210 | 0.886 | 0.905 | 0.947 |
|  | RND family efflux transporter MFP subunit | MacB | PP_4209 | 0.992 | 1.064 | 1.161 |
| 5 | Major facilitator transporter | TrpA | PP_1271 | 1.088 | 0.982 | 1.161 |
|  | Secretion protein HlyD family protein | TrpB | PP_1272 | 8.577 | 0.811 | 1.290 |
|  | RND efflux system outer membrane lipoprotein | TrpC | PP_1273 | 1.170 | 1.296 | 1.256 |
| 6 | Uncharacterized transporter HI0895 - Multidrug efflux RND transporter | UepB1 | PP_0906 | 1.143 | 0.989 | 1.007 |
|  | RND family efflux transporter MFP subunit | UepA1 | PP_0907 | 0.804 | 0.888 | 1.301 |
|  | Hypothetical protein | UepC1 | PP_0908 | 1.075 | 1.034 | 1.254 |
| 7 | RND family efflux transporter MFP subunit | MdtA | PP_3585 | 1.112 | 1.237 | 1.081 |
|  | Multidrug resistance protein | MdtB |  |  |  |  |
|  | Multidrug resistance protein | MdtC | PP_3583 | 1.332 | 0.888 | 1.149 |
|  | RND efflux system outer membrane lipoprotein | MdtD | PP_3582 | 1.012 | 0.857 | 0.841 |
| 8 | Arsenical pump membrane protein | ArsB | PP_1929 | 1.428 | 0.914 | 1.428 |
|  | Regulatory protein | ArsR | PP_1930 | 0.746 | 1.678 | 0.809 |
| 9 | RND family efflux transporter MFP subunit |  | PP_2064 | 2.939 | 3.614 | 1.109 |
|  | Acriflavin resistance protein | AcrB2 | PP_2065 | 0.700 | 6.131 | 1.900 |
| 10 | RND efflux transporter | UepA2 | PP_0179 | 0.843 | 0.703 | 1.681 |
|  | Secretion protein HlyD family protein | UepB2 | PP_0178 | 1.411 | 1.217 | 1.642 |
| 11 | RND family efflux transporter MFP subunit | AcrA | PP_5175 | 2.539 | 0.613 | 0.644 |
|  | RND family efflux transporter MFP subunit | AcrE | PP_5174 | 1.058 | 1.013 | 1.154 |
|  | Acriflavin resistance protein | AcrD | PP_5173 | 0.771 | 0.642 | 0.825 |
| 12 | Cation efflux system protein | CzcA1 | PP_0043 | 1.014 | 1.293 | 0.950 |
|  | Family cobalt/zinc/cadmium efflux transporter membrane fusion protein | CzcB1 | PP_0044 | 0.658 | 1.110 | 2.068 |
|  | Family cobalt/zinc/cadmium efflux outer membrane protein | CzcC1 | PP_0045 | 1.337 | 1.053 | 1.460 |
|  | Family heavy metal RND efflux protein (DNA binding heavy metal response regulator) | CzcR1 | PP_0047 | 1.478 | 1.400 | 1.241 |
| 13 | Acriflavin resistance protein | AcrB3 | PP_1517 | 0.237 | 1.328 | 11.214 |
|  | RND family efflux transporter MFP subunit |  | PP_1516 | 1.843 | 1.945 | 2.264 |
| 14 | Efflux transporter, RND family, MFP subunit | MexE | PP_3425 | 0.366 | 1.501 | 1.786 |
|  | Probable aminoglycoside efflux pump | MexF | PP_3426 | 0.948 | 0.883 | 0.747 |
|  | RND efflux system outer membrane lipoprotein | MexD | PP_3427 | 0.969 | 1.291 | 0.799 |
| 15 | Major facilitator transporter putative | CzcC2 | PP_2411 | 0.920 | 0.632 | 1.887 |
|  | Cobalt/zinc/cadmium resistance protein | CzcA2 | PP_2410 | 1.318 | 1.518 | 0.657 |
|  | RND family efflux transporter MFP subunit | CzcB2 | PP_2409 | 1.026 | 1.080 | 0.723 |

**Supplementary Table S7.** Bacterial secretion system-encoding gene expression profiles in the presence of 1 mM indole, 50 μg/ml ampicillin, or both 1 mM indole and 50 μg/ml ampicillin.

| **Locus_tag** | **Gene product** | **Indole 1 mM** | **Amp**  **50 μg/ml** | **Indole**  **+Amp** |
| --- | --- | --- | --- | --- |
| **Type I** | | | | |
| PP_1798 | outer membrane efflux protein | 1.187 | 1.142 | 1.386 |
| PP_4923 | outer membrane efflux protein | 1.080 | 0.515 | 0.960 |
| PP_4926 | HlyD family secretion protein | 0.717 | 0.781 | 1.496 |
| PP_4927 | toxin secretion ABC transporter protein, HlyB | 1.473 | 1.299 | 1.069 |
| **Type II** | | | | |
| PP_1046 | type II secretion pathway protein XcpQ | 1.234 | 1.834 | 1.808 |
| PP_3478 | secretion protein, putative | 1.186 | 0.883 | 1.103 |
| PP_1045 | type II secretion pathway protein XcpP | 1.060 | 0.746 | 0.772 |
| PP_1048 | type II secretion pathway protein XcpS | 1.149 | 0.921 | 0.969 |
| PP_3424 | type II secretion pathway protein XcpS | 1.147 | 1.028 | 0.820 |
| PP_1049 | type II secretion pathway protein XcpT | 0.887 | 1.379 | 1.031 |
| PP_3423 | type II secretion pathway protein XcpT | 1.582 | 0.792 | 1.215 |
| PP_3476 | secretion protein, putative | 0.563 | 1.072 | 1.930 |
| PP_3477 | hypothetical protein | 1.282 | 2.335 | 2.240 |
| PP_1050 | type II secretion pathway protein XcpU | 1.737 | 1.309 | 1.740 |
| PP_1051 | type II secretion pathway protein XcpV | 2.994 | 2.036 | 1.280 |
| PP_1052 | type II secretion pathway protein XcpW | 1.774 | 0.851 | 0.555 |
| PP_1042 | type II secretion pathway protein XcpX | 1.507 | 2.276 | 1.713 |
| PP_1053 | type II secretion pathway protein XcpY | 1.377 | 1.740 | 1.043 |
| PP_1047 | type II secretion pathway protein XcpR | 1.074 | 0.917 | 1.249 |
| PP_3483 | type II secretion system protein, putative | 0.743 | 1.185 | 1.078 |
| PP_5190 | type II secretion system protein | 0.936 | 0.951 | 0.738 |
| **Sec-SRP** | | | | |
| PP_0835 | protein-export membrane protein SecD | 1.005 | 1.067 | 1.035 |
| PP_0836 | protein-export membrane protein SecF | 1.084 | 1.259 | 1.337 |
| PP_0441 | preprotein translocase, SecE subunit | 1.228 | 1.385 | 1.330 |
| PP_0474 | Sec-dependent secretion protein SecY | 0.964 | 0.937 | 1.196 |
| PP_0834 | preprotein translocase, YajC subunit | 1.224 | 0.982 | 1.141 |
| PP_0006 | inner membrane protein, 60 kDa | 1.303 | 1.465 | 1.551 |
| PP_1345 | preprotein translocase, SecA subunit | 1.368 | 1.263 | 1.404 |
| PP_5111 | signal recognition particle receptor FtsY | 1.124 | 1.269 | 1.433 |
| PP_5053 | protein-transport protein SecB | 0.976 | 0.946 | 0.825 |
| PP_1461 | signal recognition particle protein Ffh | 1.755 | 0.207 | 1.443 |
| **Type Vb** | | | | |
| PP_1450 | activation/secretion protein, TPS family, | 1.113 | 1.243 | 1.033 |
| PP_1449 | surface colonization protein, putative | 1.023 | 0.997 | 0.993 |
| **Twin arginine targeting (Tat)** | | | | |
| PP_1041 | Sec-independent protein translocase TatA | 0.930 | 1.505 | 1.232 |
| PP_5016 | Sec-independent protein translocase TatA | 1.049 | 1.006 | 1.057 |
| PP_1040 | Sec-independent protein translocase TatB, | 0.852 | 1.007 | 1.203 |
| PP_5017 | Sec-independent protein translocase TatB | 1.112 | 1.180 | 1.009 |
| PP_1039 | Sec-independent periplasmic protein translocator | 0.912 | 0.784 | 0.700 |
| PP_5018 | Sec-independent periplasmic protein translocator | 1.189 | 0.764 | 1.159 |
| **Type VI** | | | | |
| PP_2614 | vgrG protein | 1.956 | 1.829 | 0.619 |
| PP_3106 | conserved hypothetical protein | 0.997 | 0.722 | 1.010 |
| PP_3386 | vgrG protein | 1.163 | 0.874 | 0.873 |
| PP_4049 | vgrG protein | 0.822 | 2.562 | 12.649 |
| PP_0655 | fimbrial protein-related protein | 0.904 | 0.911 | 0.829 |
| PP_2615 | hcp protein | 1.007 | 0.693 | 0.993 |
| PP_3089 | conserved hypothetical protein | 1.003 | 0.829 | 0.988 |
| PP_4082 | hcp protein | 0.953 | 1.036 | 0.663 |
| PP_4886 | conserved hypothetical protein | 0.902 | 1.068 | 0.984 |
| PP_2618 | hypothetical protein | 0.917 | 0.897 | 0.750 |
| PP_3094 | hypothetical protein | 0.934 | 0.789 | 0.930 |
| PP_4079 | conserved hypothetical protein | 1.218 | 0.506 | 1.168 |
| PP_2627 | conserved hypothetical protein | 1.208 | 1.302 | 1.256 |
| PP_3090 | OmpA domain protein | 0.960 | 0.830 | 0.879 |
| PP_3091 | conserved hypothetical protein | 1.124 | 1.117 | 1.177 |
| PP_4071 | conserved hypothetical protein | 1.377 | 0.910 | 1.133 |
| PP_2616 | conserved hypothetical protein | 0.710 | 0.798 | 1.665 |
| PP_3092 | conserved hypothetical protein | 1.175 | 1.055 | 1.250 |
| PP_3385 | conserved hypothetical protein | 1.040 | 0.689 | 0.649 |
| PP_4081 | conserved hypothetical protein | 1.292 | 2.320 | 4.320 |
| PP_3095 | chaperone-associated ATPase, putative | 0.998 | 0.827 | 1.051 |

**Supplementary Table S8.** *P. putida* KT2440 genes highly expressed in 1 mM indole and 50 μg/ml ampicillin and their homologues in *P. aeruginosa*. Susceptibility to ampicillin in the presence or absence of indole in these strains was tested as shown in Supplementary Fig. S5

| ***P. aeruginosa* mutant** | **Gene product** | **Locus**  **_tag** | **Indole 1 mM** | **Amp**  **50 μg/ml** | **Indole**  **+Amp** |
| --- | --- | --- | --- | --- | --- |
| PA5398 | oxidoreductase, FMN-binding | PP_0310 | 2.258 | 1.617 | 35.458 |
| PA2681 | transcriptional regulator, LysR family | PP_3934 | 1.752 | 1.285 | 18.557 |
| PA5534 | conserved hypothetical protein | PP_5360 | 1.097 | 0.920 | 17.066 |
| PA4126 | major facilitator family transporter | PP_3940 | 1.809 | 1.047 | 13.676 |
| PA0817 | ring-cleaving dioxygenase, putative | PP_3328 | 20.263 | 2.062 | 13.115 |
| PA1511 | vgrG protein | PP_4049 | 0.822 | 2.562 | 12.649 |
| PA1413 | transcriptional regulator, LysR family | PP_3152 | 1.662 | 1.583 | 12.060 |
| PA4353 | conserved hypothetical protein | PP_0922 | 0.620 | 1.403 | 11.853 |
| PA3676 | RND efflux transporter | PP_1517 | 0.237 | 1.328 | 11.214 |
| PA4443 | sulfate adenylyltransferase, subunit 2 | PP_1303 | 0.921 | 1.440 | 10.289 |
| PA3864 | conserved hypothetical protein | PP_3192 | 2.528 | 0.792 | 9.874 |
| PA1633 | potassium-transporting ATPase, A subunit | PP_4161 | 9.833 | 1.052 | 9.855 |
| PA5043 | type IV pili biogenesis protein PilN | PP_5082 | 1.163 | 1.342 | 7.975 |
| PA4168 | outer membrane ferric siderophore receptor | PP_0535 | 1.511 | 1.640 | 7.454 |
| PA1785 | response regulator NasT | PP_2093 | 0.754 | 3.046 | 6.586 |
| PA2556 | transcriptional regulator, AraC family | PP_2211 | 0.731 | 1.251 | 6.313 |
| PA1635 | potassium-transporting ATPase, C subunit | PP_4159 | 1.987 | 0.964 | 5.419 |
| PA5339 | endoribonuclease, putative | PP_2689 | 2.355 | 1.393 | 5.368 |
| PA2265 | gluconate dehydrogenase, putative | PP_3383 | 1.120 | 1.559 | 5.330 |
| PA4618 | xanthine dehydrogenase accessory factor, | PP_2480 | 1.589 | 0.929 | 4.900 |
| PA3592 | CAIB/BAIF family protein | PP_3393 | 1.710 | 1.024 | 4.759 |
| PA4284 | exodeoxyribonuclease V, beta subunit | PP_4673 | 3.089 | 1.015 | 4.694 |
| PA3825 | conserved hypothetical protein | PP_3435 | 1.566 | 2.432 | 4.534 |
| PA1099 | response regulator FleR | PP_4371 | 1.721 | 3.248 | 4.491 |
| PA1243 | response regulator | PP_3969 | 1.242 | 1.076 | 4.480 |
| PA1668 | conserved hypothetical protein | PP_4081 | 1.292 | 2.320 | 4.320 |
| PA5186 | alcohol dehydrogenase, iron-containing | PP_2049 | 2.132 | 1.191 | 4.216 |
| PA4172 | exodeoxyribonuclease III, putative | PP_2707 | 0.630 | 0.713 | 4.166 |
| PA2114 | major facilitator family transporter | PP_4578 | 5.069 | 1.681 | 3.999 |
| PA0247 | 4-hydroxybenzoate hydroxylase | PP_3537 | 0.713 | 0.652 | 3.980 |

**Supplementary Table S9.** Oxidative stress- or mutagenesis-related gene expression profiles in the presence of 1 mM indole, 50 μg/ml ampicillin, or both 1 mM indole and 50 μg/ml ampicillin.

| **Locus_tag** | **Gene product** | **Indole 1 mM** | **Amp**  **50 μg/ml** | **Indole**  **+Amp** |
| --- | --- | --- | --- | --- |
| PP_0915 (*sodB*) | superoxide dismutase (Fe) | 1.029 | 0.957 | 0.881 |
| PP_0946 (*sodA*) | superoxide dismutase (Mn) | 0.859 | 0.854 | 0.821 |
| PP_5309 (*oxyR*) | transcriptional regulator, LysR family | 1.029 | 1.031 | 1.107 |
| PP_2887 | catalase, putative | 1.460 | 0.385 | 0.978 |
| PP_0115 (*katE*) | catalase | 1.247 | 1.021 | 1.500 |
| PP_0481 (*katA*) | catalase | 0.599 | 0.664 | 0.586 |
| PP_3668 | catalase/peroxidase HPI | 0.538 | 0.512 | 0.390 |
| PP_2439 (*ahpC*) | alkyl hydroperoxide reductase, C subunit | 1.110 | 1.127 | 1.256 |
| PP_2440 (*ahpF*) | alkyl hydroperoxide reductase, F subunit | 0.939 | 1.924 | 1.621 |
| PP_1859 (*ohr*) | organic hydroperoxide resistance protein | 0.807 | 1.351 | 1.147 |
| PP_2060 (*soxR*) | transcriptional regulator SoxR | 1.617 | 1.446 | 1.905 |
| PP_0786 (*trxB*) | thioredoxin reductase | 1.151 | 1.249 | 1.269 |
| PP_0510 (*trx-1*) | thioredoxin | 1.081 | 1.104 | 1.023 |
| PP_5215 (*trx-2*) | thioredoxin | 1.029 | 1.365 | 1.097 |
| PP_5069 (*trxC*) | thioredoxin 2 | 0.771 | 1.108 | 0.807 |
| PP_1638 (*fpr*) | ferredoxin--NADP reductase | 0.662 | 0.373 | 0.479 |
| PP_5314 (*rubB*) | rubredoxin reductase | 1.148 | 0.990 | 1.099 |
| PP_5315 (*rubA*) | rubredoxin | 1.081 | 1.110 | 1.317 |
| PP_5371 | rubredoxin/rubredoxin reductase | 1.301 | 1.178 | 0.990 |
| PP_0944 (*fumC-1*) | fumarate hydratase, class II | 1.059 | 1.228 | 0.971 |
| PP_1686 | glutathione peroxidase | 1.219 | 7.679 | 1.334 |
| PP_1821 | glutathione S-transferase family protein | 0.750 | 0.644 | 0.751 |
| PP_2023 | glutathione S-transferase family protein | 1.218 | 2.777 | 2.073 |
| PP_2654 | glutathione S-transferase, putative | 1.160 | 1.047 | 1.266 |
| PP_3311 | glutathione-regulated potassium-proton | 1.027 | 1.574 | 0.743 |
| PP_1623 (*rpoS*) | RNA polymerase sigma factor RpoS | 1.061 | 0.986 | 1.060 |
| PP_1626 (*mutS*) | DNA mismatch repair protein MutS | 0.952 | 1.282 | 1.189 |
| PP_1629 (*recA*) | RecA protein | 0.982 | 2.614 | 2.318 |
| PP_1630 (*recX*) | RecX protein | 1.387 | 4.224 | 4.664 |
| PP_2143 (*lexA-1*) | LexA repressor | 0.500 | 1.721 | 1.154 |

**Supplementary References**

1. Kim, J., Hong, H., Heo, A., and Park, W. (2013). Indole toxicity involves the inhibition of adenosine triphosphate production and protein folding in *Pseudomonas putida*. *FEMS Microbiol. Lett.* 343, 89–99. doi: 10.1111/1574-6968.12135
2. Nelson, K. E., Weinel, C., Paulsen, I. T., Dodson, R. J., Hilbert, H., Martins dos Santos, V. A., et al., (2002). Complete genome sequence and comparative analysis of the metabolically versatile *Pseudomonas putida* KT2440. *Environ. Microbiol*. 4, 799–808.
3. Lee, Y., Pena-Llopis, S., Kang, Y. S., Shin, H. D., Demple, B., Madsen, E. L., et al., (2006). Expression analysis of the *fpr* (ferredoxin-NADP^+^ reductase) gene in *Pseudomonas putida* KT2440. *Biochem. Biophy. Res. Co.* 339, 1246–1254.
4. Jung, J., Baek, J. H., and Park, W. (2010). Complete genome sequence of the diesel-degrading *Acinetobacter species* strain DR1. *J. Bacteriol.* 192, 4794–4795. doi: 10.1128/JB.00722-10
5. Kalogeraki, V. S., and Winans, S. C. (1997). Suicide plasmids containing promoterless reporter genes can simultaneously disrupt and create fusions to genes of diverse bacteria. *Gene* 188, 69–75.
